# Epigenetic regulation in lung cancer

**DOI:** 10.1002/mco2.401

**Published:** 2023-10-26

**Authors:** Shahin Ramazi, Maedeh Dadzadi, Zahra Sahafnejad, Abdollah Allahverdi

**Affiliations:** ^1^ Department of Biophysics Faculty of Biological Sciences Tarbiat Modares University Tehran Iran; ^2^ Department of Biotechnology Faculty of Advanced Science and Technology Tehran Medical Sciences Islamic Azad University Tehran Iran

**Keywords:** aberrant hypermethylation, DNA methylation, epigenetic marker, lung cancer, tumor suppressor genes, Wnt signaling pathway

## Abstract

Lung cancer is indeed a major cause of cancer‐related deaths worldwide. The development of tumors involves a complex interplay of genetic, epigenetic, and environmental factors. Epigenetic mechanisms, including DNA methylation (DNAm), histone modifications, and microRNA expression, play a crucial role in this process. Changes in DNAm patterns can lead to the silencing of important genes involved in cellular functions, contributing to the development and progression of lung cancer. MicroRNAs and exosomes have also emerged as reliable biomarkers for lung cancer. They can provide valuable information about early diagnosis and treatment assessment. In particular, abnormal hypermethylation of gene promoters and its effects on tumorigenesis, as well as its roles in the Wnt signaling pathway, have been extensively studied. Epigenetic drugs have shown promise in the treatment of lung cancer. These drugs target the aberrant epigenetic modifications that are involved in the development and progression of the disease. Several factors have been identified as drug targets in non‐small cell lung cancer. Recently, combination therapy has been discussed as a successful strategy for overcoming drug resistance. Overall, understanding the role of epigenetic mechanisms and their targeting through drugs is an important area of research in lung cancer treatment.

## Graphical abstract

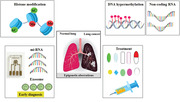

## 1 INTRODUCTION

The history of epigenetics extends from 1942, when British developmental biologist Conrad Waddington worked on *drosophila* development and genetics.^1^ He was the one who coined the term “epigenetics,”^2^ which originates from a Greek word meaning “above and beyond” (epi) the genome.^3^ He explained the association between epigenesis and gene expression in development and initiated studies on the influence of phenotypic stability in the next generation of genetic research.^4^ In this original description of the epigenetic landscape, we recognized the considerable dependency of phenotype and genotype in development and evolution.^5^, ^6^ The most impressive genome discoveries, such as chromatin structure, provide insight into a new molecular view on the definition of epigenetics more than a half‐century later. Hence, epigenetics contains mechanisms that alter gene expression without involving an underlying alteration in the DNA sequence, which can lead to phenotypic plasticity in either normal or disease states.^4^, ^7^ Identifying the basis of epigenetic mechanisms involved in biological processes can help define a better understanding of epigenetic modifications. Epigenetic mechanisms contain three main chemical modifications: DNA methylation (DNAm), histone modifications, and noncoding RNA.^5^, ^8^ Exogenous factors can influence the activity of genetic material. Gene expression is influenced by the regulation of transcription factors' accessibility to the genetic material, particularly through gene repression and/or silencing.^9^, ^10^ The epigenome is a collection of chemical modifications on DNA and histones that alter chromatin structure without any alteration in the sequence^10^, ^11^; these modifications can be heritable and reversible, besides altering gene expression.^12^, ^13^

Studies conducted during recent decades on epigenetic modifications have discovered how epigenetic traits can be influenced by chromatin structure, which directly responds to changes in phenotypic variability via regulation of gene functions. Chromatin remodeling is promoted by the interaction of epigenetic modifications. This phenomenon is attributed to the “epigenetic landscape,” which has a profound impact on cellular function and can be observed through switching genes' current expression states and turning genes on or off: DNAm, histone alterations, and RNA‐related silencing.^7^, ^14^ The regulation of significant biological processes, including a female's mammalian X chromosome inactivation, imprinting, gene silencing, and gene expression in early embryonic development, and so on.^7^, ^13^ Furthermore, epigenetics influences development and cellular differentiation.^15^

There are numerous factors that influence mammalian phenotypic variation, such as multigene effects, environmental influences, noise, and epigenetic effects that promote or repress gene transcription and expression.^16^, ^17^ The regulatory role of epigenetics in gene expression can affect the development of organs and tissues.^18^ Previous studies have shown that some of the controlling epigenetic processes like methylation of DNA, noncoding RNAs, and histone modifications might influence gene expression and are named epigenetic “marks.” All these epigenetic marks in an individual are known as the epigenome.^17^ The epigenetic marks play a significant role in the cell's processes.^19^ The stability of the epigenome in both cell divisions, mitosis, and meiosis, is fundamentally related to the development and cellular differentiation of the human life cycle.^15^ Specifically, DNAm effects on chromatin structure are notable epigenetic marks and have essential roles in development and differentiation. For instance, its relevance is evident in its effects on early development and neural differentiation.^15^, ^20^

On the other hand, these marks can be attributed to two contradictory points about normal and abnormal states.^14^ Due to this, disorders in these factors directly correlate with the prevalence of numerous human diseases, such as diverse kinds of cancer and neurological diseases.^2^ Research in epigenetics provides an advantageous interpretation of a broad range of diseases' functions and creates a meaningful repository for uncovering the main responsibility of epigenetics.^21^, ^22^ Eventually, these would be additional targets for the diagnosis and improvement of diseases like cancer. Among the various epigenetic mechanisms, it has been increasingly recognized that there is a potential relationship between abnormal DNAm and different cancers and many nonmalignant diseases.^23^, ^24^ Lung cancer is a highly common and deadly form of cancer, causing more deaths than any other form of cancer globally.^25^ It is predicted that by 2030, it will be responsible for an estimated 10 million deaths annually.^26^ Lung cancer can be grouped into two types: small cell lung cancer (SCLC) and non‐small cell lung cancer (NSCLC), based on their cell morphology. SCLC is found in 15−20% of lung cancer cases and is linked to heavy smoking.^27^ NSCLC is observed in 80−85% of lung cancers, and the five‐year survival rate after surgery depends on the tumor stage, ranging between 10 and 70%.^28^

In recent years, several small molecular inhibitors have been introduced to treat lung cancer, resulting in improved outcomes for many patients.^29^ However, some patients still do not respond well to drug therapy due to differences in their genetic, epigenetic, phenotypic, or psychosocial traits.^30^ This has led to the development of precision medicine, which aims to provide targeted, personalized treatment options.^31^ Epigenetic differences in patients are considered a key factor in the development of precision medicine,^32^ and in various types of cancer, including lung cancer, epigenetic dysregulation has been found to play a significant role in the development and heterogeneity of tumors.^33^ By targeting these epigenetic changes, precision medicine may offer more effective treatment options for individuals with lung cancer.

Over the past decade, there has been growing interest in using epigenetic changes as markers for early cancer detection. Epigenetics, which was first introduced as “soft inheritance” by Jean‐Baptiste Lamarck over 200 years ago, refers to interactions between genes and their products that create a phenotype.^34^ Epigenetic modifications may have heritability and affect gene expression and other DNA‐dependent processes without altering DNA coding.^35^ Epigenetic dysregulation is associated with many tumor types, including lung cancer and chemotherapy resistance.^36^ Unlike genetic mutations, epigenetic modifications are reversible and can be targeted by pharmacologic approaches. Remodulation of the epigenome could address tumor heterogeneity by affecting multiple signaling pathways. Lung cancer initiation and progression result from permanent genetic alterations, including point mutations, deletions, translocations, amplifications, and epigenetic modifications. These modifications impact different aspects of chromatin‐dependent processes, such as histone modifications, DNAm patterns, and microRNA (miRNA) regulation.^37^, ^38^

**FIGURE 1:**
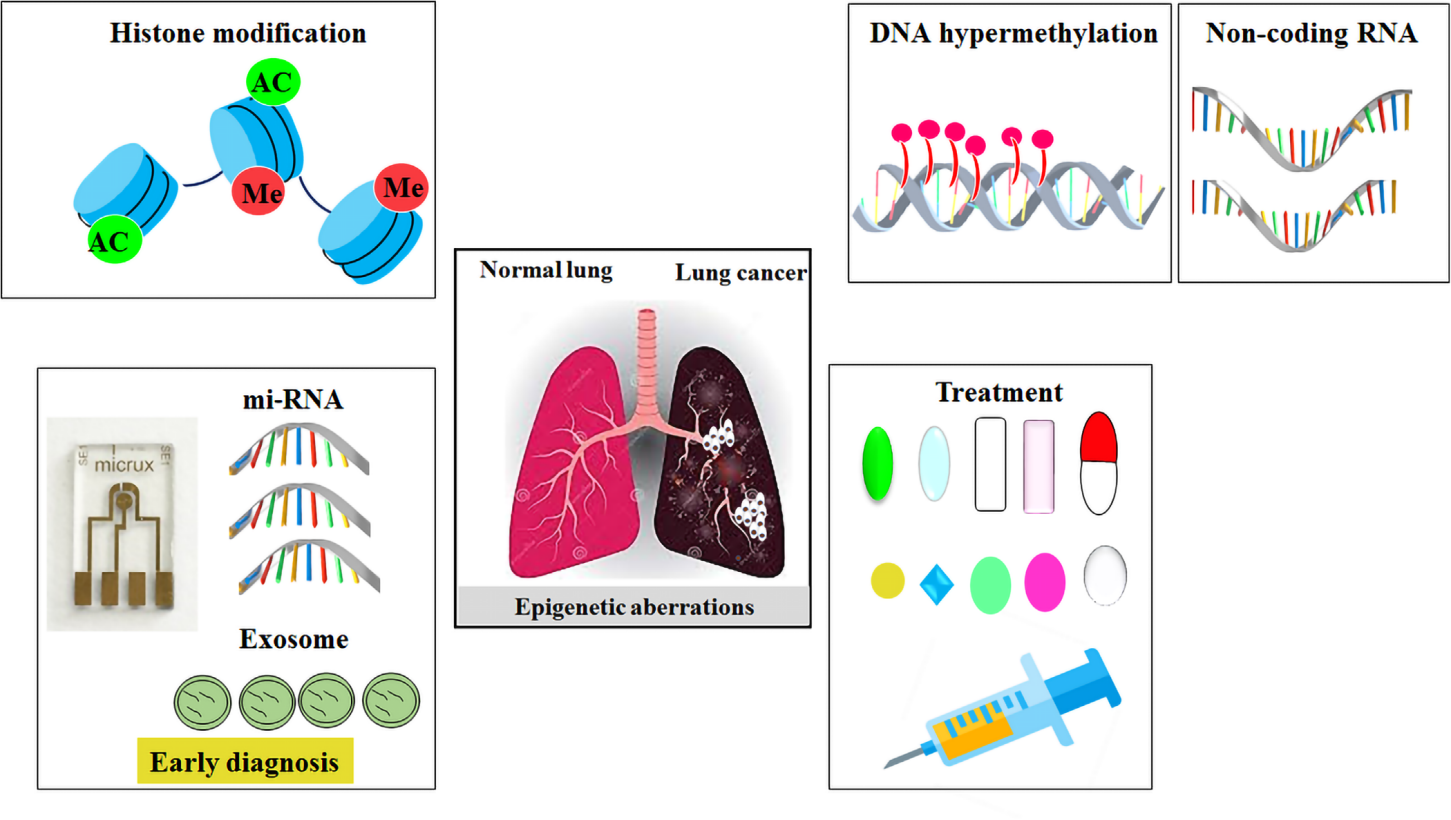
Role of epigenetics in lung cancer. Epigenetics is considered the main cause of lung cancer. Epigenetics is categorized into three phenomena: histone modification, DNA methylation, and noncoding RNAs. One of the main approaches to controlling lung cancer is early diagnosis. Micro‐RNAs and exosomes are considered reliable biomarkers to predict lung cancer at very early stages and are proposed to be considered for screening the target population.

Epigenetic regulation plays a crucial role in the development and progression of lung cancer. Epigenetic alterations, such as mutations in epigenetic regulatory mechanisms and disruptions in epigenetic patterns, have been implicated in various types of tumors, including lung cancer. In this study, we elucidated the biomarkers of epigenetics, namely DNAm, histone modification, and miRNA, and examined the epigenetic alterations occurring in these biomarkers that contribute to the development of lung cancer. Epigenetic alterations play a critical role in the initiation and progression of tumorigenesis by modulating the activation and silencing of oncogenes and tumor suppressor genes (TSGs), as well as reshaping the tumor microenvironment (TME). The abnormal proliferation and migration of lung cancer cells are orchestrated by dysregulated changes in the cancer transcriptome. However, the precise epigenetic mechanisms underlying these alterations remain unclear. Histone methylation plays a crucial role in regulating gene expression during cancer progression and metastasis. This review article specifically focuses on the abnormal hypermethylation of TSGs and its impact on tumorigenesis, as well as its influence on the Wnt signaling pathway. In summary, epigenetic regulation plays a pivotal role in the development and progression of lung cancer. Understanding the underlying mechanisms can greatly contribute to the advancement of novel therapies. Indeed, epigenetic drugs show great promise for the treatment of lung cancer. They specifically target the epigenetic modifications that occur within cancer cells, such as DNAm and histone modifications. The primary objective of these drugs is to restore normal patterns of gene expression (Figure 1). By modulating these epigenetic changes, these drugs can effectively impede tumor growth, induce apoptosis (cell death) in cancer cells, and potentially augment the efficacy of other cancer treatments. The development and utilization of epigenetic drugs represent an exciting avenue in lung cancer therapy, offering new possibilities for improved patient outcomes.

## 2 EPIGENETIC CHANGES IN LUNG CANCER

Cancer was once thought to be solely a genetic disease, but emerging evidence suggests that epigenetic modifications play a significant role in carcinogenesis.^39^ Epigenetic regulation occurs at the DNA, protein, and noncoding RNA levels. DNAm, the most studied epigenetic mechanism, leads to gene silencing.^40^ Histone proteins can be modified in a variety of ways, with some modifications promoting gene transcription. In addition, noncoding RNAs, such as miRNAs and long noncoding RNAs (lncRNAs), are recognized as epigenetic modifiers.^41^ Advances in epigenetics have improved our understanding of carcinogenesis by identifying mechanisms that alter gene expression. Epigenetic changes impact cancer hallmarks, including proliferation, invasion, metastasis, apoptosis, and regulation of the cell cycle, among others.^42^ Understanding these mechanisms may lead to the development of new therapies that target epigenetic modifications to prevent or treat cancer.^43^

### 2.1 The role of DNAm in normal and cancer cells

**FIGURE 2:**
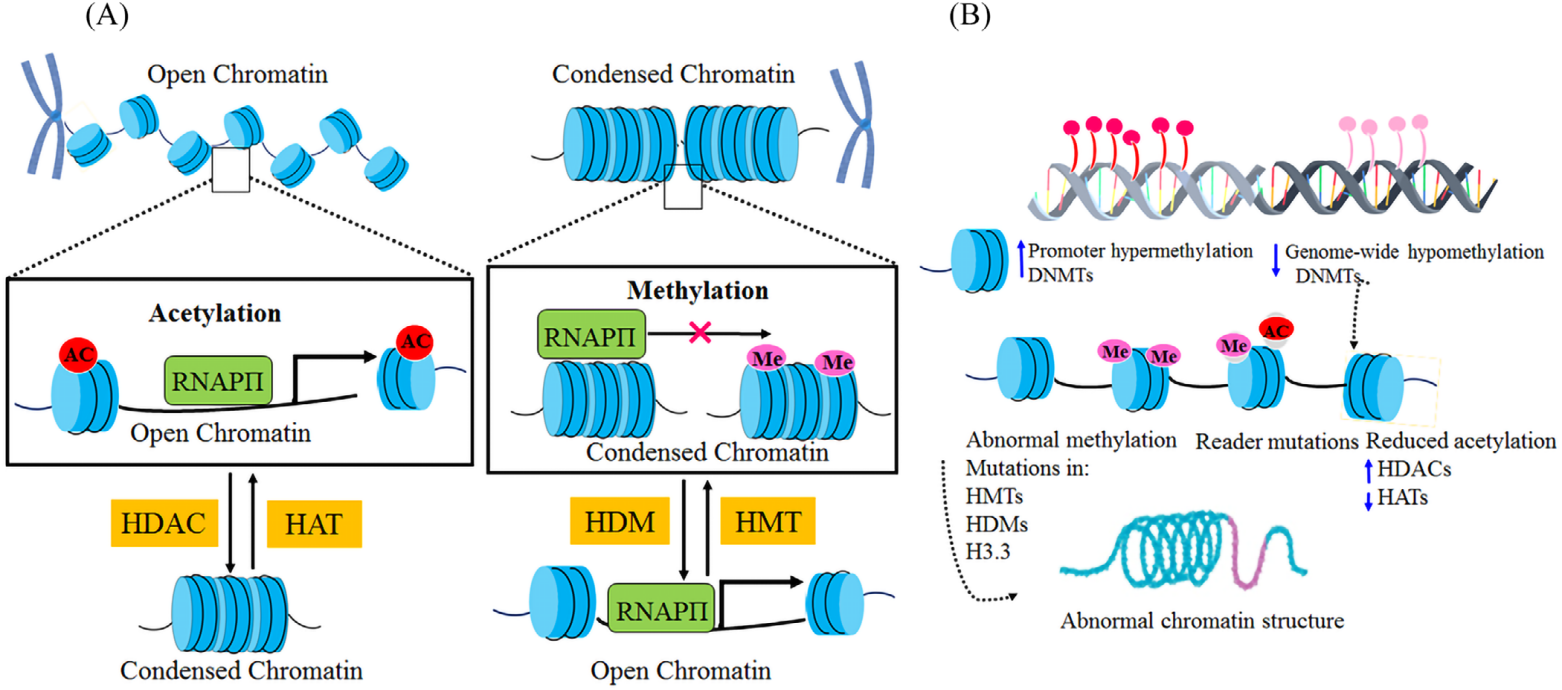
Cancer and epigenetic regulatory systems. (A) Acetylation of histones decreases their positive charge, which loosens the negatively charged DNA that is tightly wound around them. Access to the transcriptional machinery, which includes RNA polymerase and transcriptional factors, is made possible by the open chromatin structure, leading to active gene transcription. On the other hand, methylation of histones typically results in a condensed chromatin structure that makes it difficult to access the transcriptional machinery, inhibits gene transcription. Histone deacetylases (HDACs) deacetylate histones, whereas histone acetyltransferases (HATs) add acetyl groups to lysine residues on histones. Although histone demethylases (HDMs) are responsible for removing these methyl groups, histone methyltransferases (HMTs) promote the addition of mono‐, di‐, or tri‐methyl groups at arginine and/or lysine residues on histones. (B) Cancer cells exhibit both a genome‐wide loss of DNA methylation at random CpGs and hypermethylation of certain promoter CpG islands. Additionally, methyl‐binding proteins have the ability to attract HDACs. Thus, increased HMT and HDAC expression reduces chromatin access and silences Tumor suppressor gene (TSG). Aberrant epigenetic activity facilitates cancer formation and progression.

DNAm plays a significant role in repressing gene expression and maintaining genomic stability, especially by preventing recombination events between repetitive sequences.^44^ Cytosine‐guanine dinucleotides (CpG) dinucleotide islands are crucial for preserving genomic stability by promoting proper gene expression, and their dysregulation can lead to detrimental consequences for the entire genome.^45^ In cancer cells, CpG islands of TSGs are highly methylated, leading to transcriptional repression and contributing to the progression of cancer.^46^ Dysregulated cytosine methylation also occurs in other genes involved in important cellular processes such as DNA repair, apoptosis, epithelial–mesenchymal transition (EMT), cellular movement and invasion, and metastasis.^44^ On the other hand, hypomethylation of transposable element DNA can cause increased transposition within the genome, activating oncogenes and increasing chromosomal anomalies through insertional mutagenesis, leading to the development of cancer. Therefore, maintaining proper CpG methylation is critical for safeguarding against chromosomal abnormalities and guarding against carcinogenesis (Figure 2).^47^

DNAm is considered one of the most essential and influential epigenetic markers, specifically in cancer.^21^ Following the representation of DNAm and demethylation in 1969, several functions have been identified for DNAm in cellular pathways in various organisms.^5^ Although in 1975, Holliday and Pugh's^48^ study predicted that DNAm might be an epigenetic marker that involved X‐chromosome inactivation, approximately a decade later, in 1983, the study on neoplasms indicated that DNAm plays a significant role in tumor progression and the relevance of it to epigenetic modifications.^49^ Since then, DNAm has been recognized as an important epigenetic factor with long‐term, short‐term,^50^ and environmental factors.^51^ However, a wide range of studies are required to discover more about it.^23^

5‐Methylcytosine (5mC) is one of the imperative epigenetics that occurs during cellular development and diseases, and it is highly contributed to by epigenetic mechanisms.^5^ Furthermore, 6‐methyladenine may exist mostly in mitochondrial DNA and in extremely small amounts compared with 5mC. 5‐Hydroxymethylcytosine is the second most common modified base in human DNA whose biochemical and biological properties are different from 5mC.^23^ Methylation comes to pass at CpG in CpG at the 5′ positions of the cytosine pyrimidine ring by a family of DNA cytosine‐5 methyltransferase enzymes (DNMT1, DNMT3A, DNMT3B).^2^, ^3^ Although CpG dinucleotides usually occupy about ∼1% of the mammalian genome,^2^ the CpGs are scattered in CpG‐rich regions throughout the DNA sequence, which are called CpG islands.^4^, ^14^ It should be underlined that CpG islands occupy more than half of the gene promoters^14^; however, methylation can occasionally occur on CpG island shores, where the compaction rate of CpG dinucleotides is lower than that of CpG islands.

A DNA methyltransferase (DNMTs) transfers the methyl group from an adenosyl‐l‐methionine cofactor to cytosine bases that predominantly occur in CpG dinucleotides.^2^ The DNMTs are categorized into two main groups, which are de novo DNMTs and maintenance^50^ such as, respectively, DNMT3A, DNMT3B, and DNMT1.^2^ One caveat in DNAm is that, while a significant portion of the CpG island in the genome is methylated, the amount of methylation in the CpG island is lower during development and diffraction.^50^ The main function of DNAm at the specific sites of CpG is to limit transcriptional factors' accessibility to the genome and prevent transcriptional activity that impacts gene expression. This implies that methylated CpG in GC‐boxes prevented further transcription by inhibiting the binding of transcription factors.^50^, ^52^ DNAm plays an essential role in maintaining genome integrity under normal physiological conditions. Consequently, DNAm has a vital role in suppressing retrotransposons as well as genes in a tissue‐specific context to facilitate allelic expression through genomic imprinting. DNAm plays an outstanding role in major cellular processes such as development, X‐chromosome inactivation, and chromatin remodeling, for which below is an explanation.^53^

Structural studies of chromatin remodeling have revealed that any alteration in chromatin structure has a fundamental role in the regulation of gene expression patterns.^7^, ^54^ Histone modifications (particularly in tails) and DNAm are indispensable epigenetic markers for regulating gene expression levels by altering the architecture of the chromatin.^2^, ^7^, ^55^ Chromatin architecture alteration via DNAm can occur in various pathways, which play a leading role in the inhibition of transcriptional factors' attachment to DNA.^50^, ^52^ In the first place, it can impact DNA supercoiling where it is located around histone.^11^ Second, methyl CpG binding proteins, such as methyl CpG binding protein 2 (MECP2) are attached to methylated CpG by the activity of histone deacetylases (HDACs).^14^, ^50^ Because of this, transcription can be suppressed throughout the chromatin condensation process. Methyl‐CpG binding domain can be mediated by protein complexes of chromatin remodeling and histone‐modification with DNAm (methyl CpG).^56^ Chromatin remodeling can be reversible through the reversibility of DNAm or demethylation and histone modification.^50^ As a result, epigenetic modifications' reversibility can be tested to identify potential therapeutic targets, such as diseases associated with the inheritance of chromatin.^7^, ^50^

**FIGURE 3:**
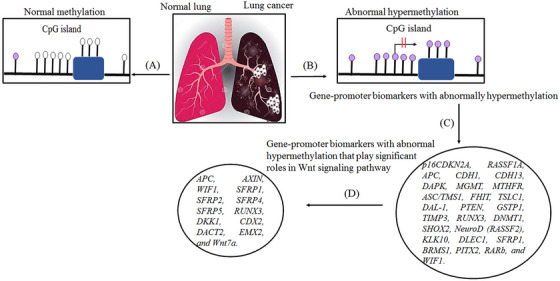
An overview of the most relevant DNA methylation (DNAm) changes found in human cancer. There are events, such as CpG‐specific DNA hypermethylation that inhibit the affected gene's activity. Hypermethylation occurs in all regions of the genome as well as in repetitive regions of the genome. The white and red circles indicate unmethylated and methylated CpG sites, respectively. Crossed‐out arrows show the transcription start site and the lack of transcription that results from DNAm. Exons are represented by green boxes, and repetitive elements by blue circle.

Epigenetic alterations, including DNAm, can lead to cancer through different mechanisms. According to previous studies, alterations in the 5mC distribution patterns can differentiate cancer cells from normal cells. There are known to be three major routes for CpG methylation that can contribute to the oncogenic phenotype.^57^, ^58^ The first is the loss of DNA cytosine methylation or general hypomethylation of the cancer genome, which leads to genome instability and increases in aneuploidy, which are both classic hallmarks of cancer. Second, focal hypermethylation at TSG promoters occurs, which causes heritable silencing and, consequently, inactivation of tumor suppressors and other genes. Third, direct mutagenesis of 5mC‐containing sequences by deamination, UV irradiation, or exposure to other carcinogens is viable. It is important that all three of these alterations happen simultaneously to contribute to cancer, suggesting that altered homeostasis of epigenetic mechanisms is central to the development of human cancer.^57^ Clearly, global DNA hypomethylation and site‐specific DNA hypermethylation have been identified in most cancers as well as in lung cancer (Figure 3).^59^, ^60^ This study detected the number of key TSGs that contain a prevalence of promoter hypermethylation in lung cancer. Abnormal hypermethylation in the promoter of classic TSGs is generally observed in cancers, a phenomenon that has been implicated in creating tumorigenesis. Genes regulating the cell cycle and DNA repairs, like *RB*, *BRCA1/2*, and phosphatase and tensin homolog (*PTEN*), have all been stated to be hypermethylated, mutated, or deleted in cancer. Furthermore, several genes exist that are seldom mutated but are silenced in cancer; promoter hypermethylation is the dominant mechanism for the loss of their functions.^61^

### 2.2 Effect of abnormal gene‐promoter hypermethylation in lung cancer

Lung cancer is the number one cause of cancer and is the second leading cause of cancer‐related deaths in the United States, which threatens people's health around the world.^62^ More deaths are caused by lung cancer than breast, prostate, colorectal, or brain cancers combined.^63^ There are over 1.5 million new cases of lung cancer every year, making it the world's costliest cancer.^62^ In Iran, cancer‐related mortality is caused primarily by lung cancer; therefore, it rates second and third in male and female mortality, respectively.^64^ The most common type of lung cancer is NSCLC, which accounts for more than two‐thirds of lung cancer cases,^63^ and in 85% of cases, lung cancer is NSCLC.^62^ A majority of cases of NSCLC are lung adenocarcinomas (LUAD) (40%), followed by lung squamous cell carcinomas (LUSC) (25%), and small cell carcinomas, which represent 10% of all NSCLC.^62^ Furthermore, SCLC accounts for approximately 15% of all lung cancers^65^ and is characterized by high proliferative rates, a strong tendency to metastasize early, and poor prognoses.^66^

Cell proliferation, resistance to growth suppression and cell death, angiogenesis, invasion, and metastasis are hallmarks of cancer that are largely controlled by genes and epigenetic changes. The epigenetic changes play a significant role in contributing to tumorigenesis and contain missense mutations, copy number variations, insertions, deletions, and recombination of DNA.^53^ During carcinogenesis, genes can become activated via mutational activation of oncogenes or inactivation of TSGs underpins that result in the formation of cancer. Thus, we can say that the recognition that heritable changes, regulated by epigenetic alterations, may also be vital for the evolution of all human cancer types. Additionally, it has been discovered that oncogenic traits may be accumulated via epigenetic disturbances.^57^ Epigenetic mechanisms play a significant role in maintaining heritable changes in gene expression potential and chromatin organization over generations. Thus, genetically identical cells develop distinct phenotypes due to epigenetic regulation of transcription.^53^

DNAm plays a vital role in normal mammalian development. The roles of DNAm in the regulation of gene expression patterns that were initially thought to be associated with gene silencing have become increasingly apparent over the past few decades. However, studies of the function of genes mutated in humans with disease patterns characterized by abnormal DNAm have provided some of these insights.^67^ Researchers reported the first evidence of aberrant DNAm in cancer tissues almost 40 years ago, when they estimated tumors of various cancer types had lower levels of DNAm than normal tissue samples. Southern blotting techniques were used to detect these changes at the level of specific genes or repetitive sequences. In addition to the global loss of 5mC, the hypomethylation of CpG islands was first reported in the calcitonin gene in the mid‐1980s, which was related to cancer disease.^68^ A schematic illustration of hypermethylation and hypomethylation in repetitive regions of the genome is shown in Figure 3. Aberrant DNAm leads to several diseases, like cancer, neurodegenerative diseases, immune disorders, atherosclerosis, and diverse age‐associated diseases. On the other hand, aberrant hypermethylation or hypomethylation in DNA contributes to being considered markers for cancer formation and tumor progression^23^ and to date, the role of altered DNAm patterns in cancer development has been extensively analyzed.^57^, ^59^ In this review, our primary objective is to elucidate the most common epigenetic alterations and abnormal DNAm patterns observed in the development of lung cancer. Specifically, we will concentrate on exploring the phenomenon of aberrant hypermethylation in various genes that is associated with lung cancer. By focusing on these specific gene targets, we aim to provide a comprehensive understanding of the role of DNAm in lung cancer pathogenesis. According to previous studies, abnormal DNAm in cancer diseases.^69^ Recent extensive studies have reported that the different patterns of methylation in several types of diseases would be aided by finding the potential contribution of hypermethylation and hypomethylation in specific genes that are involved in various cancer diseases.^2^, ^23^

Epigenetic alterations are proven to influence tumorigenesis. Abnormal DNA hyper‐ and hypo‐methylation is capable of contributing to various types of cancer in regions rich in CpG sites, particularly the methylation of TSGs.^23^, ^70^ In contrast to DNA hypermethylation, DNA hypomethylation mainly occurs less frequently than methylation in repeated DNA sequences.^23^ Abnormal methylation in noncancerous and cancerous diseases could be caused by an increase or decrease in DNAm in a specific region of the genome.^23^ Likewise, the determination of hyper‐ and/or hypo‐methylation in diseases can also be used as a biomarker and would help to find the cause of diseases and individual risk factors, particularly for diseases that are related to hypermethylation,^23^, ^71^ like melanoma.^72^ The hypermethylated FMR1 promoter is identified as a biomarker for the diagnosis of fragile X syndrome.^2^, ^23^ Hypermethylation can drive several types of diseases and disorders, and together with that, the increased methylation in promoters’ TSGs has a potential contribution to the development of tumors and carcinogenesis.^23^, ^71^ Methylation is the most prevalent epigenetic means of inactivating promoters.^73^ As an example, hypermethylation is considered a primary event in melanoma and/or a result of CpG islands. Hypermethylation in certain promoters correlates with TSGs. Thereby, following these processes can lead to inhibiting the activity of the antioncogene genes in the tumor, which is called CpG island methylation or phenotype (CIMP), particularly in the lung, melanoma, colorectal cancers (CRCs), and so on.^23^, ^72^

Interestingly, hyper‐ and hypo‐methylation patterns on a particular region of the gene promoter have distinct behaviors in diseases, resulting, for example, in two completely different neurological diseases. However, the same process occurs in cancer.^2^, ^23^ On the other hand, a specific gene with hyper‐ and/or hypo‐methylation can cause a similar type of disease. For example, the level of hypomethylation of glutamate decarboxylase 1 (*GAD1*) in Parkinson's disease is higher than in healthy individuals, and/or the DNA hypermethylation of norepinephrine is higher in Parkinson's disease.^3^ DNA hypomethylation associated with lung cancer may increase genomic instability. Abnormal hypermethylation in promoters can lead to the silencing of genes involved in pathways with hallmarks of cancer, such as DNA repair, cell cycle regulation, promotion of apoptosis, or control of key tumor‐relevant signaling networks.^53^ In cancer, changes in DNAm patterns and aberrant methylation of promoter CpG are important mechanisms for suppressing TSGs and an effective tool for developing molecular biomarkers.^74^ As shown by recent developments in molecular genetics studies.^75^ However, it is still unclear how extensive and sequence‐specific DNA hypermethylation is in cancer.

**FIGURE 4:**
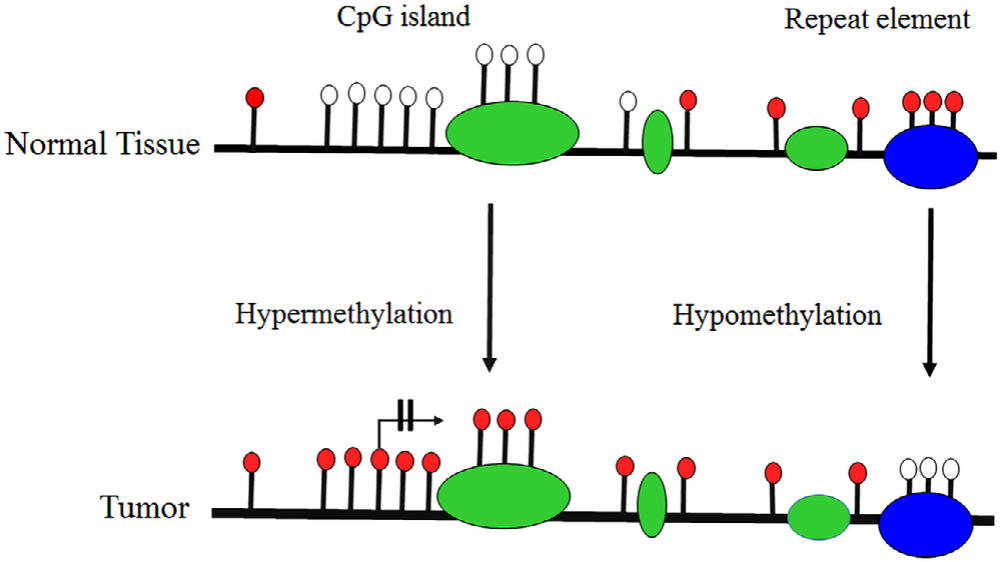
Hypermethylation in gene promoters (A) Hypermethylation in gene promoters, in normal cellular function, plays a significant role in regulating gene promoter expression. (B) Abnormal hypermethylation occurs in gene promoters, particularly in cancer cells, which can result in the silencing of Tumor suppressor genes (TSGs) and contribute to the uncontrolled growth and division characteristic of cancers, such as lung cancer. (C) Hypermethylation of some genes’ promoters is a common feature of lung cancer and has been identified as a potential diagnostic and prognostic biomarker as well as a therapeutic target. (D) Additionally, hypermethylation of certain genes in the Wnt signaling pathway has been identified as a biomarker for lung cancer. Dysregulation of this pathway, which plays a role in cell proliferation and differentiation, has been linked to lung cancer and various types of cancer. Hypermethylation of genes in the Wnt pathway can alter gene expression and contribute to cancer development and progression.

If diagnosed and treated at an early stage, the mortality rate of this disease can be greatly reduced. Researchers have recently suggested the use of aberrant CpG island methylation for early detection of lung cancer, and tumor‐suppressor genes are considered a molecular marker system in the promoter region for early identification of lung cancer, for example, cyclin‐dependent kinase inhibitor 2A (*p16INK4A*), O^6^‐methylguanine‐DNA methyltransferase (*MGMT*), and retinoic acid receptor beta (RARB).^76^ Promoter hypermethylation is known as a significant mechanism for silencing tumor‐suppressor genes in cancer and can be used for molecular biomarker development.^76^ In the 1990s, hypermethylation of CpG islands was reported for several known TSGs and other genes involved in important growth control,^74^ including apoptosis, cell adherence, DNA repair, and cell‐cycle control.^77^ Aberrant DNAm provides another mechanism for the inactivation of TSGs along with the genetic mechanisms that promote lung cancer occurrence and progression. Approximately 70% of known genes harbor CpG islands within their transcription start site. In lung cancer, Ras association domain family 1A (*RASSF1A*) and cyclin‐dependent kinase inhibitor 2A (*CDKN2A/p16*) are the most commonly epigenetically inactivated TSGs.^78^ Aberrant methylation of cytosine at the promoter regions of genes is one of the major mechanisms of the downregulation or upregulation of genes in lung cancers. An increasing number of genes have been intensively investigated for their methylation status in lung cancers (as shown in Figure 4 and Table 1).^74^, ^77^, ^79^, ^80^, ^81^, ^82^, ^83^ The methylation of genes has been shown to be associated with the smoking history of patients with lung cancer. In LUAD and squamous cell carcinomas, the frequency of *p16*, *MGMT*, *RASSF1*, death‐associated protein kinase (*DAPK*), methylenetetrahydrofolate reductase (*MTHFR*), adenomatous polyposis coli (*ACP*), glutathione S‐transferase pi gene (*GSTP1*), Cadherin 1 (*CDH1*), and *PTEN* promoter methylation was significantly higher among smokers than never‐smokers.^84^, ^85^ The main focus of this article, described in this section, is the characterization and role of DNA hypermethylation in lung cancer and, in particular, its effect on TSGs in their promoters, which are known as epigenetic markers in lung cancer.

**TABLE 1:** A list of genes with abnormal hypermethylation in lung cancer and their biological functions. NSCLC, non‐small cell lung cancer; SCLC, small cell lung cancer.

Gene names	Biological function	Reported histological type	References
Retinoic acid receptor β (RARβ)	Cell differentiation and proliferation	NSCLC, SCLC	^201^
Cysteine aspartyl‐specific proteases (CASP8)	Apoptosis	SCLC	^202^, ^203^
Fragile histidine triad protein (FHIT)	Cell proliferation and apoptosis	NSCLC, SCLC	^204^
DNA mismatch repair protein Mlh1 (MLH1)	DNA repair	NSCLC	^205^
MutS homolog 2 (MSH2)	DNA repair	NSCLC	^205^
Phosphatase and tensin homolog (PTEN)	Cell cycle regulation	NSCLC	^206^
Runt‐related transcription factor 3 (RUNX3)	TGF‐β/Wnt signaling pathway	NSCLC, SCLC	^207^
Semaphorins 3 B (SEMA3B)	Cell adhesion and regulation of cell motility and cell adhesion	NSCLC, SCLC	^208^, ^209^
Short Stature Homeobox 2 (SHOX2)	Cell differentiation and proliferation	NSCLC, SCLC	^210^
Transforming growth factor beta receptor II (TGFBR2)	Inhibition of epithelial cell growth	NSCLC	^211^
Telomerase reverse transcriptase (TERT)	Immortalization of cancer cells	Lung cancer	^212^
Tumor necrosis factor receptor superfamily member 6 (TNFRSF6)	Apoptosis	SCLC	^202^
TRAIL‐R1/DR4	Apoptosis	SCLC	^202^
Tumor suppressor in lung cancer 1 (TSLC1)	Cell adhesion	NSCLC, SCLC	^80^, ^211^
Methylenetetrahydrofolate reductase (MTHFR)	DNA synthesis and remethylation reactions	NSCLC	^85^, ^213^
Target of methylation‐induced silencing 1 (ASC/TMS1)	Innate immune response and apoptosis	NSCLC	^214^
Erythrocyte membrane protein band 4.1‐like 3 (DAL‐1)	Regulation of cytoskeleton	NSCLC	^80^, ^215^
TIMP metallopeptidase inhibitor 3 (TIMP3)	Invasion and metastasis	NSCLC	^201^
Ras Association Domain Family Member 2 (RASSF2)	By inhibiting activated RAS signaling, acts as a tumor suppressor	NSCLC	^216^, ^217^
Ras Association Domain Family Member 2 (NORE1A/ RASSF5)	Involved in proapoptotic and kills cells in a Ras‐dependent manner	NSCLC	^91^, ^218^
Ras protein‐specific guanine nucleotide‐releasing factor 2 (RASGRF2)	Involved in H‐Ras signaling	NSCLC	^75^
Kallikrein‐related peptidase 10, also known as normal epithelial cell specific gene 1 (NES1) (KLK10 / NES1)	Tumor suppressor gene (TSG) and subgroup of serine proteases	NSCLC	^77^, ^219^
Deleted in lung cancer protein 1 (DLEC1)	Cell cycle regulation	NSCLC	^80^, ^220^
Breast cancer metastasis suppressor 1 (BRMS1)	Suppresses metastasis	NSCLC	^221^
Paired‐like homeodomain transcription factor 2 (PITX2)	Transcription factor	NSCLC	^222^
Transforming growth factor‐β (TGFBI)	Cell adhesion and migration	NSCLC, primary lung cancer	^223^

NSCLC, non‐small cell lung cancer.

#### 2.2.1 Ras association domain family 1 isoform A (*RASSF1A*)

*RAS* (a small GTPase) oncogene families are the most commonly activated oncogenes in human diseases, and mutations in these oncogenes cause a variety of cancers.^86^ Multiple mitogenic pathways are activated by *RAS* upon activation by its well‐documented effectors, *RAF*, *PI3K*, and *RalGDS*.^87^ Furthermore, excessive *RAS* stimulation can also promote apoptosis or senescence, but it is much less well understood how these pathways lead to cell death. The six core RASSF family proteins, which contain conserved Ras association domains, can function as Ras effectors.^88^ All *RASSF* proteins lack enzymatic activity and appear to function as scaffolding and localization molecules that allow multiple pathways to communicate and sometimes compete with each other.^87^ The *RASSF* family of *RAS* effectors, notably *RASSF1*, is known to mediate many of these effects.^87^ The RASSF plays significant roles in cell apoptosis, genomic and microtubule stability, and cell cycle regulation, and it is coded for two main transcripts, RASSF1A and RASSF1C, by alternative splicing.^89^

*RASSF1A* is located within a 120‐kb region of chromosome 3p21.3 and is known as a frequent target for aberrant methylation in lung cancer. On chromosome 3p21.3, loss of heterozygosity (LOH) is one of the most common and earliest events in the development of lung cancer^76^ and approximately 90% of SCLC and 50−80% of NSCLC have allele loss in this region.^76^ *RASSF1A*, as a *RAS* effector, not only crosslinks *K‐RAS* with proapoptotic signaling pathways, such as Bax and Hippo, but also facilitates other signaling pathways for *RAS* and DNA repair, like inflammation, autophagy, protein acetylation, and ubiquitination.^88^ The tumor suppressor *RASSF1A* or RASSF1A (as a tumor suppressor) inhibits Ras‐induced tumorigenesis by inducing apoptosis after hyperactivation of *RAS*.^87^ *RASSF1A* encodes a tumor suppressor that inhibits the *RAS→RAF→MEK→ERK* pathway and inactivates genes in human cancers.^90^

*RASSF1A* forces a cell cycle arrest by inhibiting the activity of cyclins A and D1, and it interacts with *CNK1*, *MST1*, *Salvador*, and *MOAP1*, which may modulate apoptosis.^75^, ^90^, ^91^ Further studies reveal that the hypermethylation of the *RASSF1A* promoter is another factor contributing to this gene's reduced expression, along with the LOH.^75^ The inactivation of *RASSF1A* is implicated in the development of many human cancers and is inactivated by gene deletion, point mutations, or transcriptional silencing by inappropriate promoter methylation.^91^ There has been evidence that both alleles of the *RASSF1A* promoter are hypermethylated, causing the gene to lose expression, and *RASSF1A* is known to be inactivated in many human tumors.^91^ Thus, *RASSF1A* is considered a TSG that has been widely explored in lung cancer and other malignant tumors.^75^, ^87^

#### 2.2.2 O^6^‐methylguanine‐DNA methyltransferase

*MGMT* is a protein that has been shown to interfere in DNA repair, and it is believed that *MGMT* gene silencing or a lack of synthesis is what causes *MGMT* deficiency.^92^ By eliminating adducts from the O^6^ position of guanine, it can shield cells against the effects of alkylating chemicals. The therapeutic effects of alkylating drugs are, therefore, blunted by high levels of *MGMT* activity in cancer cells, which can be a key factor in treatment failure.^93^ Loss of *MGMT* activity is caused by the epigenetic silencing of *MGMT*, which is achieved by methylating certain CpG islands of its promoter in tumor tissues of different malignancies, including lung tumors.^94^

The simplest method for repairing chemically damaged DNA involves an enzyme‐catalyzed reversal of the chemical reaction that produced the changed base in the first place. As a result, a DNA‐alkyltransferase is able to eliminate methyl and ethyl adducts from the O^6^ position of guanine, reestablishing the base's natural structure. The fact that the *MGMT* gene is silenced by promoter methylation in about 40% of gliomas and colorectal tumors, as well as in about 25% of NSCLCs, lymphomas, and head and neck cancers, suggests the significance of this enzyme, *MGMT*, as a DNA alkyltransferase, in the development of certain types of human tumors.^95^ The *MGMT* suppressor gene encodes a DNA repair protein that clears alkyl groups from guanine's O^6^ position.^96^ The *MGMT* gene is epigenetically silenced by the methylation of particular CpG islands in its promoter, which results in a lack of *MGMT* enzyme production.^97^ *MGMT* expression in tissue can be determined by immunohistochemistry, and *MGMT* promoter methylation status can be determined by polymerase chain reaction (PCR). Cytology or microbiopsies can be used to perform these experiments.^98^ *MGMT* protein expression fluctuates, with tumor tissue expressing *MGMT* proteins at a lower level than normal tissue.^94^ 1160 tumor and 970 control samples of either plasma, serum, or bronchoalveolar lavage fluid were examined for the presence of *MGMT* promoter methylation. The likelihood that the MGMT gene promoter is methylated was greater in tumor tissue compared with control samples (OR = 4.43, 95% CI: 2.85–6.89), indicating that NSCLC frequently has *MGMT* gene promoter methylation. Adenocarcinoma and squamous cell carcinoma have distinct molecular profiles.^99^ *MGMT* promoter methylation, as assessed by PCR, was seen in a prospective series of NSCLC patients as well as in healthy controls.^100^ With an overall value of 18%, the promoter methylation frequency varied from 0 to 50%. The presence of *MGMT* promoter methylation in the sputum of smokers who have never developed cancer points to a link between promoter methylation and the risk of developing lung cancer.^101^ Additionally, compared with healthy controls, patients with lung cancer had lower levels of *MGMT* protein expression in their bronchial epithelium, indicating a possible link between *MGMT* expression and the risk of developing lung cancer.^102^ More research is required to determine whether *MGMT*‐expression is a signal for early lung cancer detection.^103^ This could be a field of pre‐neoplastic alterations for the recurrence of NSCLC.^104^ When malignant and nonmalignant lung tissues from the same individuals were compared, many discrepancies were found where the methylated genes in the nonmalignant tissues were not methylated in the corresponding tumor tissue. In three surgical series, the predictive value of *MGMT* promoter methylation was examined. According to this research, 15−51% of patients had positive *MGMT* promoter methylation results. The promoter methylation status of the *MGMT* gene was reported by Brabender et al.^105^ in 34 out of 90 (38%) resected NSCLC samples and in 16 out of 90 (18%) matched normal tissue. *MGMT* promoter methylation in matched tumor tissue always occurred in conjunction with *MGMT* promoter methylation in normal tissue. Patients who did not have *MGMT* promoter methylation fared considerably better than those who did, indicating that this condition may be a predictive biomarker for NSCLC's more aggressive nature.^105^

#### 2.2.3 P16INK4a

The involvement of *p16* among the proteins and elements contributing to cancer development and tumor cell growth has to be considered. *P16* is a crucial TSG that may help regulate the cell cycle, and it frequently exhibits CPG promoter hypermethylation in different human cancers.^106^ On chromosome 9 (9p21.3), the *P16INK4a* gene, often referred to as the *CDKN2A* gene, is crucial for controlling the cell cycle, senescence, apoptosis, cell invasion, and angiogenesis.^107^, ^108^ More than 70% of cell lines obtained from all histologic categories of human non‐small cell lung tumors have been inactivated. Inhibiting the retinoblastoma protein (pRb) pathway via downregulating cyclin‐dependent kinases is its primary function in cell cycle regulation. In contrast, suppression of the *p16* gene results in the phosphorylation of pRb, which unblocks the cell cycle and causes unchecked cell growth and enhanced proliferation in all cancer types.^109^ A number of genetic changes, such as homozygous deletions, promoter hypermethylation, point mutations, and LOH, can cause *p16* to become inactive. Its dysregulation is caused by homozygous deletions and promoter hypermethylation, whereas point mutations and minor deletions, especially missense mutations, change the structure and activity of *p16*.^110^ As a result, several transcriptional factors and oncogenes promote *p16* dysregulation. The activation of other antioncogenes, such as *p21*, may make up for the genetic inactivation of p16. This shows that an imbalance between the activation of oncogenes and oncoproteins and their suppression leads to carcinogenesis. When *p16* is downregulated, cancer progresses, but when it is overexpressed in certain solid tumors, the prognosis is poor.

A higher prevalence of *p16* hypermethylation has been observed in a number of human malignancies, including NSCLC, where *p16* methylation is present in about 40% of lung tumors.^111^ Additionally, promoter hypermethylation is linked to numerous pathways as well as a 22−60% reduction in *p16* expression.^112^ Only four out (18.1%) of the 22 instances of *p16* gene hypermethylation discovered in tumor tissues showed hypermethylation status in normal tissues, indicating that tumor cells exhibit a higher *p16* hypermethylation frequency than nearby normal cells.^113^ Additionally, it was shown that the *p16* gene is an important genetic target for the etiology of lung cancer in smokers.^114^ When compared with nonsmokers, the promoter region of p16 appears to be substantially more frequently affected by smoking status.^115^ Additionally, a striking correlation was found between smoking features like length of smoking or time since stopping and *p16* methylation, which may help explain the increased prevalence of NSCLC.^116^

#### 2.2.4 Death‐associated protein kinase (*DAPK*)

*DAPK* (also termed *DAPK1*) a 16‐kDa tumor suppressor, constitutes one of the CaM‐regulated kinase superfamilies that relate to Ser/Thr kinases.^117^ The *DAPK* gene is situated on chromosome 9q34.1, which is divided into three domains: a repeat domain, a kinase domain, and a death domain.^117^, ^118^ *DAPK* is best known for its involvement in a variety of cellular processes such as cytoskeletal‐associated protein kinase, inflammation, autophagy, and as an essential mediator of apoptosis‐inducing pathways via TRAIL, Fas, THN‐α, and IFN‐γ.^118^, ^119^ However, hypermethylation of CpG islands in the *DAPK* promoter region effectively impairs its ability to induce apoptosis and disrupt the cell cycle, resulting in the initiation of carcinogenesis.^120^ Emerging evidence has revealed the involvement of *DAPK* activity and inactivity in neurodegenerative diseases such as Alzheimer's disease, stroke, neuronal death, and cancer.^119^

In more than two decades of empirical research into cancer, abnormal *DAPK* promoter methylation has been reported in more than 30 types of human cancers due to impaired expression of *DAPK*, including breast, leukemia, and lung cancers, although the status of methylation significantly differs from type to type.^119^, ^121^ The promoter methylation prevalently occurs in aggressive cancer cases, for instance, lung cancer, which often contains DAPK methylation in the promoter region, such as NSCLC, and allelic loss of the DAPK gene is commonly observed in both NSCLC and SCLCs cell lines.^121^, ^122^, ^123^ Based on this discovery, it was determined that DAPK functions mainly as a TSG.^119^, ^121^

As a result of *DAPK* silencing due to abnormal methylation status in lung carcinoma, highly metastatic clones were deficient in the expression of *DAPK*. According to these results, NSCLCs, but not non‐neoplastic lung tissues, were detected to contain high levels of methylated *DAPK*.^124^, ^125^ On the basis of lung cancer patient samples, hypermethylation of DAPK1 was detected in 39% of cancerous tissues; additionally, high levels of hypermethylation of DAPK1 were also found in 33% of NSCLC tissues.^126^ In comparison with precancerous lung cells, NSCLC frequently has higher methylation levels of *DAPK*. According to tumor‐node‐metastasis (TNM) stages, the rate of *DAPK* methylation may differ at each stage.^123^ The proportion of *DAPK* promoter methylation is higher in lymph node metastasis NSCLC patients compared with those without the metastasis.^118^, ^123^ It is evident that NSCLC cases often have a higher *DAPK* level, which leads to a decrease in their likelihood of a 5‐year survival rate compared with patients without methylated *DAPK*. On average, 58.1% of tumor tissues from NSCLC contain methylated *DAPK*.^123^ “Around 50% of *DAPK1* was hypermethylated in NSCLC”.^127^ In this regard, prior studies revealed that the abnormal methylation of *DAPK* is exhibited in both adenocarcinomas and squamous cell carcinomas, and it should be noted that *DAPK* promoter hypermethylation can serve as a notable abnormal indicator of NSCLC early stage; as another example, it appears that methylated *DAPK* in alveolar hyperplasia of LUAD points to a role of *DAPK* influence during adenocarcinoma early development.^128^, ^129^ The findings of numerous studies show that *DAPK* is frequently hypermethylated in NSCLC tumors, but hypermethylated *DAPK* has also been detected in about one‐third of all cases of SCLC.^122^, ^130^ Accordingly, *DAPK* promoter hypermethylation can therefore be used as an indicator of tumor progression in NSCLC cases, which means that *DAPK* methylation may have a significant correlation with the prognostic feature of NSCLC as well as the origin of NSCLC.^119^, ^123^ In general terms, *DAPK* methylation can serve as an early biomarker for both diagnosis and prognosis in patients with NSCLC, such as those identified in NSCLC patients' sputum samples and/or serum samples.^119^

#### 2.2.5 Adenomatous polyposis coli (*APC*)

As a homodimer protein, *APC* is predominantly found within the nucleus and cytoplasm of cells.^104^ The *APC* gene consists of two promoters (1A and 1B), situated on chromosome 5q21‐q22, which are responsible for encoding a protein of around ∼311 kDa.^131^, ^132^ Transcription‐initiating regions are started by distinct codons in two exons (1A and 1B), which results in multiple transcripts based on alternative splicing.^132^ *APC* contains a C‐terminal domain referred to as the “basic domain,” which is responsible for interacting with microtubules in microtubule‐nucleating activity.^104^, ^133^ Traces of *APC* tumor suppressor activity are detected in both the nucleus and cytoplasm, indicating its antioncogenic role, particularly with regard to Wnt signaling canonical pathways.^134^ *APC's* association with Wnt signaling pathways is one of the main factors that indicates its importance as a tumor suppressor.^133^ *APC* is an integral part of the Wnt signaling antagonizing genes, which in the nucleus once it interacts with β‐catenin and downregulates it through its negative regulation of the canonical Wnt pathway (Wnt/β‐catenin pathway) abolishes tumor growth and progression and consequently, promotes apoptosis and inhibits proliferation.^134^, ^135^ Any *APC* malfunction can have an impact on the apoptosis pathway and facilitate tumorigenesis.^135^, ^136^ Besides being an antagonist of the Wnt signaling pathway, *APC* performs many functions, including counteracting tumorigenesis via inhibition of tumor invasion and progression, participating in cell migration and adhesion, promoting differentiation, transcriptional activation, apoptosis, and involvement in cell survival throughout development.^133^, ^135^, ^136^ Evidence suggests that *APC* participates in chromosomal segregation during various phases of mitosis, such as the regulation of the mitotic spindle and/or kinetochore localization, thereby contributing to cell polarity and its directional migration.^133^, ^135^ However, DNA replication activity is prevented by *APC*s binding directly to DNA.^133^, ^135^ Hypermethylation of CpG islands in the *APC* gene promoter region dramatically abrogates its expression, which causes the chromatin conformation to change drastically and disrupts aberrant transcription factor binding to CBF (CCAAT‐binding factor).^137^ Multiple cancer types have been found to have an abnormally methylated *APC* promoter, an indication that there might be a causal link between *APC* gene promoter 1A methylation and cancers including breast, lung,^132^ and prostate cancer.^138^ Based on research, *APC* is related to lung cancer types (sporadic and familial), which are thought to suffer from a higher incidence due to an allelic loss.^127^ It should be mentioned that a direct correlation exists between methylated *APC* and the first oncogenic event.^139^ In specimens of biopsies and serum from early‐stage lung cancer patients, higher levels of *APC* gene methylation were detected than in healthy individuals (nonmethylated *APC* gene), and there may be a correlation between advanced lung cancer stages and hypermethylation of the *APC* gene.^120^ Furthermore, sputum specimens from patients with lung cancer also contained high levels of methylated *APC* in NSCLC in comparison with adjacent noncancerous tissue.^140^ There is also a link between cancer and mutations in the *APC* gene. For instance, studies on CRC have revealed that mutations in the *APC* gene also cause tumorigenesis in either a direct or indirect manner.^131^, ^135^ Hypermethylation of *APC* promoter 1A particularly in the NSCLC cell line, causes its specific transcript to be silenced.^132^, ^141^ Both NCLC and SCLC have been hypermethylated in the *APC* promoter 1A (NSCLC 53%, SCLC 26%).^132^ Based on other studies, this rate can be as high as 96% of primary lung carcinomas that exhibit promoter abnormal methylation.^142^ As a consequence, most of the primary NSCLCs contain abnormally methylated *APC* gene promoters, which may serve as a biomarker of primary lung cancer.^132^ According to the investigation, the primary stage I lung tumors contain *APC* promoter hypermethylation.^143^ Researchers evaluated *APC* as a biomarker for early diagnosis of stage I/II NSCLC using plasma samples. Therefore, NSCLC plasma samples containing methylated *APC* were detected in around 20% of samples that presented tumor‐specific hypermethylation. As a result, APC could serve as a potential epigenetic biomarker for diagnosing NSCLC with 90,0% specificity and 47.27% sensitivity.^77^ Among lung cancer patients, the extent of *APC* promoter methylation appeared to be related to lymph node status (nodal status) and cancer stage (T stage), particularly in smoking samples, and positive *APC* hypermethylation in NSCLC patients was considerably linked to a longer survival rate in comparison with those lacking it, therefore suggesting that it may be a valuable indicator of patient survival.^144^ Despite this, prior studies suggested that hypermethylation of *APC* promoters contributed to inferior survival for patients with advanced NSCLC.^141^ In light of these findings, the repressive function of *APC* in cancer would provide an attractive therapeutic target for lung cancer by boosting its function and using it as a biomarker.^77^, ^131^

#### 2.2.6 Glutathione S‐transferase pi gene (*GSTP1*)

Glutathione S‐transferases (GSTs) are indispensable enzymes that facilitate the detoxification of both endogenous and exogenous toxins by binding them with glutathione. GSTP1 is a standout member among its counterparts in the GST family.^145^ These enzymes affect the signaling pathways involved in cell division, proliferation, and apoptosis by their interactions with various elements (such as regulatory kinases). Consequently, GST has cytoprotective and regulatory properties and contributes significantly to the proliferation and demise of cancer cells.^146^ Progression, recurrence, and growth of tumors are frequently impacted by changes in epigenetic regulatory systems, such as promoter hypermethylation.^147^ Numerous tumor types, including neuroblastoma, hepatocellular carcinoma (HCC), endometrial, breast, and prostate cancers, are regularly affected by *GSTP1* methylation, which is frequently linked to tumor formation or a bad prognosis.^148^ Cytoplasmic, mitochondrial, and microsomal GSTs are the three main protein subfamilies that have been found to be active glutathione transferases.^149^ Microsomal GSTs are membrane‐associated proteins involved in the metabolism of eicosanoids and glutathione.^150^ The largest subfamily of these transferases, cytoplasmic GSTs, performs special functions. They have thiol transferase activity, reduce trinitroglycerin, dehydroascorbic acid, and monomethyl decanoic acid, and catalyze the ethyl maleate and 5 3 isomerization of ketosteroids. They also catalyze the thiolysis of 4 nitrophenyl acetate.^151^ The following seven subtypes of GSTs are distinguished based on similarities in amino acid sequences, variations in gene structures, and immunological cross‐reactivity: alpha (α), pi (π), mu (μ), theta (θ), omega (ω), sigma (σ), and zeta.^152^

##### *GSTP1*: A major regulator in the occurrence and development of cancer

Among the GST family members, GSTP1 has garnered the most extensive research attention. Chromosome 11q13 is the location of the *GSTP1* gene (π). It was initially identified from a cosmid library, has nine exons, and measures 3.2 kb in length. It shields cells from cytotoxins and cancer‐causing agents.^153^ The gene is approximately 3 kb long and has six introns. The 5′ end of the gene has significant G + C and CpG content, which is typical of *Hpa*II microfragment islands. In humans, *GSTP1* comprises two identical dimeric subunits, each with 210 amino acids and two G and H binding sites, and is usually found in pairs. Various G and H sites with various amino acid residues in GST may have diverse functions. The interaction between GST amino acid residues, GSH thiols, and common electrophiles is catalyzed by *GSTP1* at the H site, which is particularly bound to GSH or GSH analogues.^145^ There are many physiological roles for *GSTP1*: It breaks down a variety of carcinogenic substances, detoxifies and eliminates potentially genotoxic foreign complexes, and defends cells from DNA deterioration and cancer development. Early research on the GST family showed that the *GSTP1* gene is crucial for a number of physiological functions, such as catalysis and deoxylation of electrophilic chemicals, the control of oxidative stress, cell signaling, and the development of cancer.^145^ *GSTP1* effectively defends cells against cancer‐causing and electrophilic substances. According to various studies*, GSTP1* shields cells against oxidants and electrophile‐caused genomic damage.^154^ The metabolism of various chemotherapeutic drugs and apoptosis resistance are both regulated by *GSTP1*. It has been discovered that platinum‐based medications are processed by *GSTP1*, which enables *GSTP1* to be expressed in ovarian cancers. As a result, *GSTP1* could be exploited as a target gene and potential response biomarker for platinum‐based anticancer chemotherapy. Additionally, the metabolism of cisplatin and carboplatin in ovarian cancer cells is greatly influenced by *GSTP1*.^155^

##### *GSTP1* methylation: a tissue biomarker that performs well in several types of malignancies

The GSTP1 gene's promoter region is typically methylated, and variations in methylation status reduce normal gene expression, which may impair or eliminate the detoxification and antioxidant effects of the gene. The *GSTP1* gene is hypermethylated in a number of cancer types. A major tissue biomarker, *GSTP1*, is effective in detecting a variety of cancers, including PCa, breast, lung, and HCC.^156^ Recent studies have verified that hypermethylation of *GSTP1* inactivates the *GSTP1* gene and is a significant contributor to liver cancer. It may enhance the risk of HCC and is strongly linked to a bad outcome for HCC patients.^157^ Hypermethylation of the *GSTP1* gene promoter region has been suggested as a possible biomarker for separating HCC from other liver conditions. The *GSTP1* gene promoter's methylation may be related to how invasive HCC is. *P16* inactivation caused by *GSTP1* methylation may be due to chronic hepatitis B virus infection.^158^ Acute chronic hepatitis B liver failure is characterized by *GSTP1* methylation and oxidative stress‐induced liver damage. Acute hepatitis B liver failure also exhibits abnormal GSTP1 promoter methylation, which may be highly prognostic of short‐term death. Consequently, GSTP1 may be a possible predictive biomarker of acute liver failure linked to acute hepatitis B.^159^ The methylation of GSTP1 has a significant impact on liver illnesses and may be used to treat those diseases. GSTP1 methylation is related to the prognosis and recurrence of PCa and may be an epigenetic diagnosis marker.^159^ In early breast cancer events, GSTP1 hypermethylation also happens. A significant barrier to DNAm study of the GSTP1 gene is its heterogeneous DNAm pattern, which accounts for part of the conflicting discrepancies in the involvement of GSTP1 promoter methylation in breast cancer.^160^ While prior research has not conclusively linked *GSTP1* methylation to the clinicopathological features of PCa, it is linked to a more aggressive ER‐positive breast cancer phenotype.^161^ Furthermore, ER positivity is linked to *GSTP1* methylation. It was discovered that the clinicopathological features of breast cancer were linked with *GSTP1* methylation.^162^ As a result, *GSTP1* methylation is crucial for the study of breast cancer. In NSCLC patients, the frequency of *GSTP1* methylation in cancer tissues ranges from 0 to 25%, while neighboring benign tissues show less or no methylation. The development of neuroblastoma may be aided by abnormal *GSTP1* methylation, which might also be utilized as a novel diagnostic tool. Additionally, methylation of the *GSTP1* gene is linked to acromegaly's resistance to somatostatin analogue therapy.^163^ As a result, *GSTP1* methylation seems to be important in a number of disorders.

##### Phosphatase and tensin homolog

Recently, the gene *PTEN* deleted on chromosome 10 (*PTEN*; also known as MMAC/TEP1), a novel potential tumor suppressor, was discovered and located on chromosome 10q23.3.^164^ *PTEN* is a dual phosphatase that may bind to tyrosine and serine‐threonine sites. *PTEN* modifies a crucial pathway regulating cell proliferation and survival by blocking the activation of Akt/protein kinase B via phosphatidylinositol 3, 4, 5‐trisphosphate.^165^ The PTEN protein's tumor suppressor activity depends on its COOH‐terminal region.^166^

Proteins lacking the COOH terminal region are produced by mutations in *PTEN* exons 7, 8, and 9. Due to the quick degradation of these truncated products, *PTEN* protein expression is lost. A number of neoplasms, particularly those of the central nervous system, thyroid, breast, prostate, and bladder, as well as those of endometrial origin, have been linked to genetic changes at the *PTEN* locus. Eighty percent of patients with Bannayan‐Zonana syndrome, Cowden's disease, and juvenile polyposis have germline mutations of *PTEN*, indicating that *PTEN* is also an inhibitor of intestinal polyposis.^167^ Somatic *PTEN* hypermethylation has been identified as a mechanism of *PTEN* downregulation in a subset of malignancies such as prostate cancer, colon cancer, and endometrial carcinoma. Epigenetic alterations play an important role in cancer progression through hypermethylation and silencing of TSGs.^168^ Notably, *PTEN* promoter methylation and other epigenetic processes, such as loss of mRNA and protein expression, have both been linked to the activation of AKT and other phosphatidylinositol 3‐kinase pathway effectors and may play a role in the development of melanoma.^169^ Recently, a mechanism of functional loss in some endometrial malignancies was discovered: promoter methylation of PTEN.^168^

#### 2.2.7 *PTEN*‐mediated regulation of the metabolic pathway

The PTEN/PI3K pathway may have an impact on crucial metabolic processes during cell proliferation and cancer. *PTEN* is engaged in the regulation of metabolic pathways through PI3K‐dependent and independent actions, according to recent results from two independently developed transgenic mouse models based on two identical *PTEN*‐containing bacterial artificial chromosomes.^170^ Garcia‐Cao et al. showed that transgenic mice overexpressing *PTEN* have smaller sizes as a result of fewer cells, higher energy consumption, and less body fat accumulation. These mice's cells exhibit reduced absorption of glucose and glutamine, elevated levels of mitochondrial oxidative phosphorylation, and resistance to oncogenic transformation. The literature is unclear on PTEN's contribution to insulin‐stimulated glucose absorption. Considering PTEN's capacity to modify insulin signaling, there is strong evidence that it plays a part in the regulation of glucose absorption.^171^ Nakashima et al. have demonstrated that *PTEN* overexpression in adipocytes inhibits insulin‐stimulated, *PI3K* activation‐dependent 2‐deoxyglucose uptake and glucose transporter type 4 (GLUT4) translocation, a crucial stage in insulin signaling that ultimately results in reduced glucose cellular uptake.^172^

##### Posttranscriptional regulation

In many cancers, miRNA helps control the expression of PTEN. In fact, it has been shown that in some malignancies, such as hepatocellular, ovarian, and lung cancer, the oncogenic miR‐21, one of the most frequently upregulated miRs in cancer, directly targets and suppresses PTEN expression.^173^ Recent research has demonstrated that miR‐25 regulates PTEN levels in human cancers and aids in the development of experimental malignancies.^173^ MiR‐25 presents another intriguing connection between the MEK/ERK and PI3K/PTEN/AKT/mTOR pathways, as was mentioned above for the contribution of c‐Jun. Recent studies have resulted in the competing endogenous RNA (ceRNA) theory, which postulates that both noncoding and protein‐coding genes have a novel, mRNA‐dependent noncoding role that allows them to act as a ruse to counteract the effects of particular miRs on other RNA.^174^ This seems to be the case for PTEN pseudogene 1 (PTENP1), which exhibits high levels of sequence similarity to PTEN mRNA in areas with micro RNA target sites. PTENP1 was also discovered to control PTEN expression by trapping PTEN‐targeting micro RNA, lengthening PTEN mRNA half‐lives, and raising PTEN protein levels.^175^

##### PTEN in lung cancer

Except for LUSC, where PTEN is mutated in 6−9% of cases and profoundly altered in up to 15% of cases (taking into consideration loss of expression as well), PTEN mutations are uncommon in NSCLC and SCLC.^176^ Other methods to reduce PTEN expression and function may be significant in lung cancer, given that the loss of PTEN protein expression is reported in 24−44% of cases.^177^

#### 2.2.8 Cadherin family genes

Cadherin family genes encode calcium‐dependent membrane proteins involved in vertebrates' cell–cell adhesion. Therefore, they comprise the intercellular junctional complex as well as participate in tissue morphogenesis, cell physiological function, particularly functional structures, including organ epithelia, and coordinated cell movement.^178^, ^179^, ^180^ The cadherin superfamily contains two main subfamilies: classical cadherin and nonclassical cadherin.^178^, ^181^ They are divided into three main families: the major cadherin (CDH), the protocadherin (PCDH) family, and the cadherin‐related (CDHR) family.^178^ In spite of their involvement in cell aggregation and tumor suppression, any dysfunction and/ or downregulation in their expression leads to their being implicated in tumor invasion and metastasis.^182^ The classical group of cadherins was initially discovered by separate research groups in the 1980s. Over a hundred members of the cadherin superfamily have been explored in humans to date.^178^ The classic cadherins have two main subfamilies. Type I includes CDH1, CDH3, CDH2, CDH4, CDH15 (E‐, P‐, N‐ and R‐, M‐ cadherins, respectively), and as well, type II classic cadherins include 7D cadherins, desmosomal cadherins, Flamingo, and CELSR cadherins.^178^, ^182^

##### Cadherin 1 (*CDH1*)

Cadherin 1 (*CDH1*), also called E (epithelial cadherin), belongs to the type‐I classical cadherins subfamily of the major cadherin (CDH) family, which is a highly conserved transmembrane glycoprotein family.^178^, ^183^ The CDH1 TSG situated on 16q22.1 was responsible for transcribing a protein of 120‐kDa.^183^, ^184^ Since the discovery of *CDH1* in the late seventies, E‐cadherin has been identified as a fundamental component of epithelial cell–cell adhesion, cell polarity, cell proliferation, EMT, and epithelial phenotype maintenance that prevents cells from moving by keeping them together.^179^, ^183^, ^185^ E‐cadherin was found to participate in many signaling pathways, including β‐catenin/Wnt pathway, the Hippo pathway, and growth factor receptor tyrosine kinase (*RTK)/EGFR/MAPK* pathway, *P‐120/Rho/RAC* pathway, and the Src family kinase signaling pathways.^183^, ^185^

Any alteration in *CDH1* gene expression is implicated in several cancer‐related signaling pathways, such as the Wnt signaling pathway, which has been identified in diverse cancer types.^180^, ^185^ For instance, aberrant *CDH1* promoter methylation has been reported in many cancer types in humans, such as gastric, leukemia,^186^ breast, and lung cancers.^187^ Lack of *CDH1* expression results in loss of contact inhibition and cell polarity, metastases, invasion, tumor proliferation, activation of motility, and migration, which also correlate with the development and progression of primary tumors and^183^, ^185^ tumor dedifferentiation.^188^ In breast cancer cells, *CDH1* expression was lost due to promoter hypermethylation. In light of the aberrant methylation of its 5′ CpG island, it is related to the progression and development of ductal breast carcinoma.^179^, ^187^ In spite of *CDH1* being absent in cancer cells, studies have indicated that restoring E‐cadherin expression can suppress tumor progression and invasion.^99^, ^185^

It has been more than 20 years since CDH1 and CDH13 methylation were identified in lung cancer,^99^, ^189^ so more insight has been gained into the involvement of both genes’ methylation in lung cancer. CpG islands of the *CDH1* promoter were identified as being hypermethylated in NSCLCs and SCLCs.^99^, ^190^ According to a study, the overall frequency of *CDH1* promoter hypermethylation was detected in 67.1% of NSCLC patients.^186^ In primary resected NSCLCs, a high frequency of *CDH1* methylation was detected at the promoter region in 18% of the samples, whereas it was not found in the nonmalignant samples from the same patients.^191^ According to a later study, in 80% of NSCLC samples, CDH1 hypermethylation was detected, although it was also detected in 14% of the surrounding histologically noncancerous lung tissue. Furthermore, 63% of NSCLC tissues were identified to contain high levels of *CDH1* hypermethylation.^126^ Even though declining levels of E‐cadherin cause metastasis to form, its expression in tumors causes them to be less invasive due to their cell‐to‐cell adhesion, including in NSCLC.^188^ Hypermethylated *CDH1* was found to be significantly linked with longer overall survival.^186^ Promoter hypermethylation of *CDH1* was detected in plasma cell‐free DNA samples of lung cancer patients.^184^ Thus, *CDH1* gene expression has a critical role in the progression of lung cancer and can be a valuable independent, favorable prognostic factor, resulting in its expression, which may prove useful for treatment in NSCLC patients.^188^

##### Cadherin 13 (*CDH13*)

*CDH13* (cadherin 13, also called H (heart)‐cadherin and T‐cadherin) belongs to cadherins without a cytoplasmic domain in nonclassical cadherins.^182^, ^192^, ^193^ As a result of its structural differences and truncated nature, CDH13 is classified independently from classical cadherins.^192^, ^194^ The *CDH13* TSG is situated on 16q24.2−3.^189^ Although H‐cadherin has neither cytoplasmic nor transmembrane regions, it binds to the cell's surface membrane through a glycosylphosphatidylinositol anchor, and it plays a role in cell–cell adhesion.^192^, ^194^ H‐cadherin was first described in the embryonic nervous system,^193^ and it was also detected that it is expressed in several tissues, including the heart, cardiovascular system, kidney, lung, and particularly neural tissues.^192^

For instance, *CDH13* hypermethylation in the promoter region has been reported in several types of cancer, including, CRC, pituitary adenoma, and breast cancer.^189^, ^195^ The downregulation of *CDH13* due to hypermethylation seems to exist prevalently in invasive cancers.^196^ In addition, hypermethylated *CDH13* results in the loss of its expression, which is related to metastasis, invasiveness, and tumor malignancy.^189^, ^195^ As well as hypermethylation of *CDH13*, promoter CpG islands have been detected in both NSCLC and SCLC.^190^ Furthermore, it has been identified in LUAD.^196^ NSCLC primary tumors exhibit higher levels of aberrant *CDH13* promoter methylation than SCLC cell lines, which is accompanied by the silencing of *CDH13* gene expression.^189^ In the late nineties, *CDH13* hypermethylation at the promoter 5′ region was found in 45% of primary lung cancers, which is responsible for *CDH13* gene inactivation.^197^

Methylation of *CDH13* leads to inactivation, which correlates with tumorigenicity in NSCLC. Previously, the frequency of methylated *CDH13* was detected in 66% of the tumor samples.^198^ Based on lung cancer patient samples, hypermethylation of *CDH13* was detected in 57% of cancerous tissues although it was also detected in 2% of the surrounding histologically noncancerous lung tissue. In addition, high levels of hypermethylation were found in 47 % of NSCLC tissues.^126^ Consequently, *CDH13* has higher levels of methylation in NSCLC.^126^ The methylation of the *CDH13* promoter in patients with stage I NSCLC who underwent surgery with the intention of curing the cancer was more likely to recur early.^199^ There is evidence of hypermethylation of the CDH13 promoter in 34% of CDH13 gene aberrant methylation involved in lung cancer pathogenesis.^189^ Simultaneous hypermethylation of *CDH1* and *CDH13* was detected in 10.2% of primary NSCLC samples, and both of these hypermethylations were associated with longer overall survival.^200^ bronchial lavage samples.^84^ In tumor and sputum samples of patients with NSCLC, aberrant promoter hypermethylation of *CDH13* was detected.^198^ Consequently, *CDH13* promoter hypermethylation was present in 23% of serum samples from NSCLC patients, but not in healthy individuals' serum samples.^198^ Based on plasma analyses, the combination of three methylated genes, *CDH13, APC*, and *RASSF1A* in patients with NSCLC was found to provide high sensitivity and specificity (71.82 and 80%, respectively) in plasma for NSCLC diagnosis. Besides, *CDH13* promoter aberrant methylation has been found to have high sensitivity (33.64%).^77^ The *CDH13* promoter methylation status in NSCLC tumor tissue and NSCLC plasma samples was identified. Tumor‐specific hypermethylation occurred at a significantly higher frequency in NSCLC tissues and plasma samples than in normal tissues and cancer‐free plasma.^77^ It has been suggested that detecting methylated *CDH13* in bronchial lavage, serum, and plasma may serve as a biomarker for noninvasive early detection of NSCLC.^84^, ^198^ A summary of the respected genes is illustrated in Table 1.

### 2.3 Advances in Wnt signaling pathway related with DNAm in lung cancer

The Wnt signaling pathway is an evolutionarily conserved pathway that governs numerous cellular functions during both embryonic and adult stages. Under normal developmental conditions, Wnt signaling is responsible for controlling several aspects of development and is involved in different physiological processes, particularly adult stem cell maintenance, cell proliferation, cell fate determination, apoptosis, cell migration, and cell polarity. However, the Wnt signaling pathway plays a fundamental role in embryonic development, orchestrating the formation of embryonic organs and structures. In addition, this pathway has significant roles in normal adult homeostasis, motility, polarity, and stem cell renewal.^135^, ^137^ The Wnt signaling system is a highly intricate network that encompasses two primary pathways: the canonical Wnt/β‐catenin (Wnt/β‐catenin dependent pathway) and the noncanonical Wnt/β‐catenin pathway (β‐catenin‐independent pathway) that are identified to control both physiological and pathological processes, including cancer. The noncanonical Wnt/β‐catenin pathway was further dispensed into two additional branches, the Wnt/planar cell polarity (Wnt/PCP) and the Wnt/calcium pathways (Wnt/Ca^2+^). Both of them are involved in cancer development and dissemination.^224^, ^225^ The Wnt/β‐catenin pathway entails the migration of β‐catenin to the nucleus, where it activates target genes by means of TCF/LEF (T‐cell factor/lymphoid enhancer factor) transcription factors.^226^ Conversely, the noncanonical pathways, operate independently of the β‐catenin‐T‐cell factor/lymphoid enhancer‐binding factor (TCF/LEF). This pathway primarily controls cell polarity and migration. As a consequence, the self‐renewal of certain mammalian tissues is significantly influenced by Wnt signaling. The Wnt signaling pathway is a fundamental mechanism that plays a critical role in several biological processes, including lung tissue repair and metabolism, development of the hematopoietic system, hair follicle renewal, liver metabolism and regeneration.^226^ These essential functions underscore the importance of Wnt signaling in maintaining tissue homeostasis and suggest that dysregulation of this pathway may contribute to the pathogenesis of various disorders, including cancer, cardiovascular diseases, bone disease, and neurodegenerative disorders.^226^ Wnt signaling is one of the critical cascades controlling normal development and physiology and has been developed to perform diverse functions in cancer. In several types of cancer, the aberrant Wnt signaling pathway has a complicated role in cancer development, such as promoting proliferation and differentiation of cancer cells, which can have a direct impact on tumorigenesis and can be considered potential targets for cancer treatment.^227^, ^228^

**FIGURE 5:**
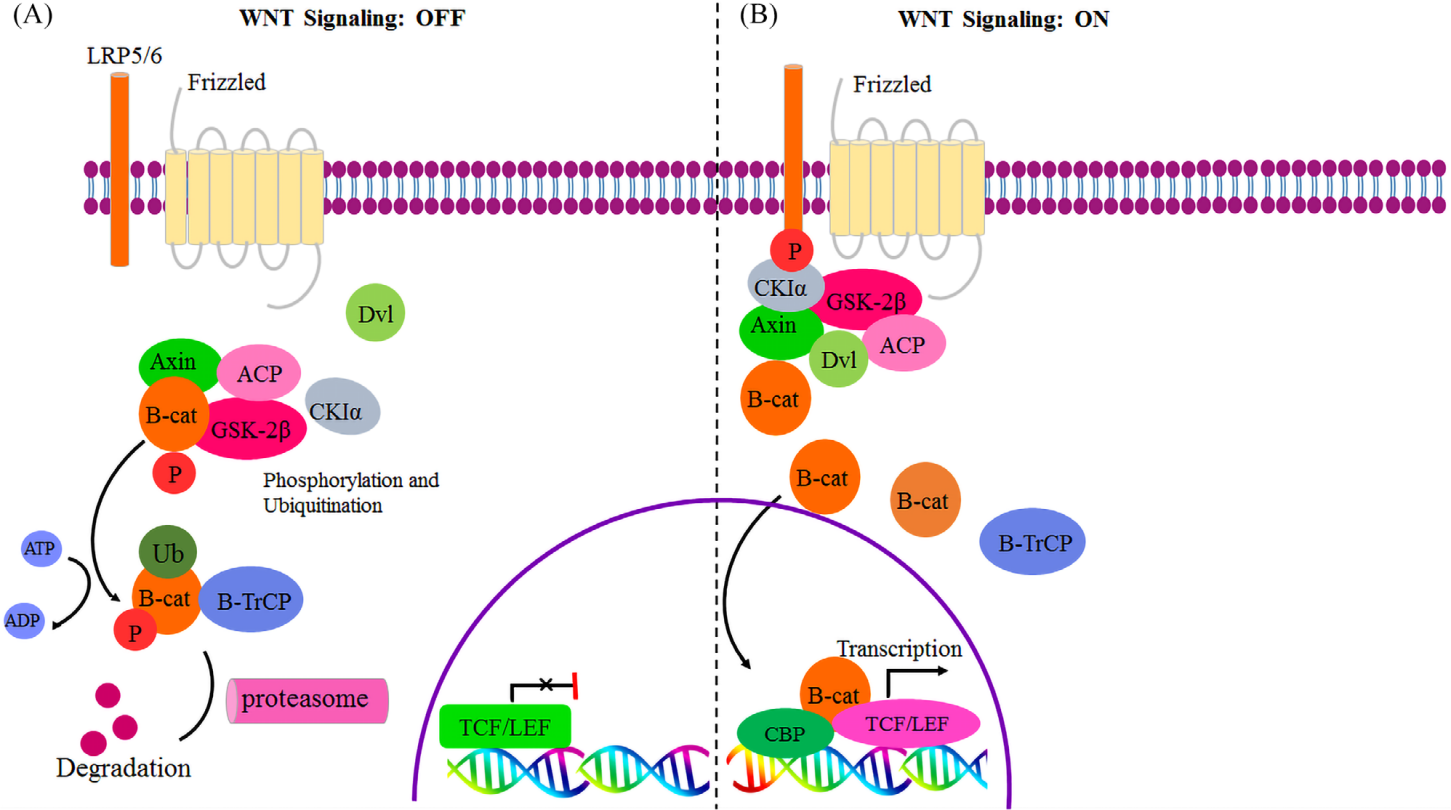
Schematic representation of the Wnt signaling canonical pathway, which is a common one. (A) “Wnt OFF state”: When Wnt is off state, the β‐catenin destruction complex binds to β‐catenin and is phosphorylated by glycogen synthase kinase‐3β (GSK3β) and CK1. During the next steps, β‐catenin gets phosphorylated, which in turn stimulates two processes. β‐Catenin is ubiquitinated by β‐transduction repeat‐containing protein (β‐TrCP) via attachment to specific residues, which ultimately leads to β‐catenin destruction by the proteasome. As a result, if β‐catenin is destroyed, it cannot enter the nucleus and function as a transcription factor, resulting in transcriptional inhibition of target genes. (B) “Wnt ON state”: Wnt binds to its receptor and coreceptor, which are called frizzled receptor and LPR6/5, respectively. β‐catenin destruction complex (axis inhibition protein (Axin), adenomatous polyposis coli (APC), casein kinase 1α (CK1α), and GSK3 β) is the main component of the Wnt pathway. Upon binding, the receptors are activated. As a result of receptor activation and phosphorylation, leading to recruitment of dishevelled (DVL) and all components of the complex. Subsequently, the lipoprotein‐related protein 5/6 (LPR6/5) coreceptor inactivates and immobilizes the β‐catenin destruction complex. Then GSK3β and casein kinase 1 (CK1) interact with LPR6/5, which are unable to bind β‐catenin and thus cannot phosphorylate it. When β‐catenin is not phosphorylated, it can enter the nucleus and be bound to the transcription factor namely the T‐cell factor/lymphoid enhancer factor family (TCF/LEF). TCF/LEF is actually bound in conjunction with β‐catenin which turns the transcription of the target genes on.

Numerous malignancies, for instance, lung, colon, breast, and prostate cancers, exhibit overexpression of Wnt proteins, specifically Wnt1, Wnt2, Wnt3A, and Wnt5A which act as oncogenic activators for the canonical Wnt signaling pathway. This pathway, in turn, has been shown to promote self‐renewal of cancer stem cells (CSCs). Consequently, recognizing the particular Wnt proteins that regulate the canonical Wnt signaling pathway and CSCs could offer significant potential targets for the development of personalized cancer therapies.^229^, ^230^, ^231^ The Wnt signaling pathway is inhibited by a number of proteins that are also known as Wnt antagonists, including the dickkopf (*Dkk*) family, the secreted frizzled‐related proteins (*SFRPs*) proteins, dishevelled (*DVL*), Wnt inhibitory factor (WIF‐1), disabled 2 (*Dab2*), and Cerberus.^232^ In the off state of the Wnt pathway, there is a complex of proteins known as the β‐catenin destruction complex, which includes axis inhibition protein (*AXIN*), *APC*, casein kinase 1α (*CK1α*), and glycogen synthase kinase‐3β (*GSK3β*) respectively. β‐catenin is destroyed by this destruction complex via phosphorylation, ubiquitination, and ultimately proteasome degradation. Thus, during the Wnt off state, no β‐catenin should remain, and there should be a low level of β‐catenin (shown in Figure 5).^232^ The Wnt/ β ‐catenin pathway is linked to both cancer and noncancer diseases. Hence, lung, heart, liver, bone, and neurological disorders are all accompanied by dysregulation of the Wnt/‐catenin pathway.^226^ Consequently, the Wnt/β‐catenin pathway route is up to the intracellular concentration of β ‐catenin level; as a result, it is elevated in malignancies and significantly influences angiogenesis, invasion, proliferation, and apoptosis.^139^ Wnt signaling has switched from an off to an on status in several types of cancer as a result of Wnt inhibitor downregulation. Therefore, aberrantly activating the Wnt pathway, especially Wnt/β‐catenin, promotes the development of CSCs, invasion, deterioration, metastasis, and resistance to chemotherapy.^227^, ^229^, ^233^

Indeed, dysregulation of the Wnt signaling pathway, due to genetic or epigenetic alterations has been implicated in numerous cancers. In this regard, there is ample evidence that gene promoter hypermethylation of a pivotal protein, specifically Wnt antagonists/inhibitors, involved in Wnt signaling pathways is crucial to the development of several types of cancer,^234^ such as pancreatic, colorectal, gastric, and lung cancer.^229^, ^234^, ^235^ Hence, the critical significance of Wnt antagonists/inhibitors in modulating the Wnt signaling cascade and their consequential impact on cancer progression and prognostic implications are underscored. The Wnt pathway plays a critical role in the pathogenesis of acute lymphoblastic leukemia (ALL). Abnormal promoter methylation of seven Wnt inhibitors (secreted frizzled related protein 1 (*sFRP1*), *sFRP2*, *sFRP4*, *sFRP5*, *WIF1*, Dickkopf‐3 (*DKK3*), and human homologue of dapper (*HDPR1*) is linked to the activation of the Wnt pathway in ALL resulting in the downregulation of the expression of these inhibitors. This hypermethylation is observed in ALL‐derived cell lines and bone marrow mononuclear cells from ALL patients. Aberrant methylation‐mediated downregulation of these inhibitors is significantly associated with an adverse clinical outcome in ALL, manifested by a substantial reduction in both 10‐year disease‐free survival and overall survival rates, thereby highlighting the potential prognostic value of abnormal Wnt signaling in this disease.^236^

Furthermore, it has been observed that hypermethylation of certain Wnt inhibitors, namely *runt‐*related transcription factor 3 (RUNX3), *DKK‐3*, and sFRP1, is positively associated with the recurrence of esophageal squamous cell carcinoma (ESCC). This highlights the potential significance of hypermethylation of the promoter regions of Wnt antagonists/inhibitors as a predictive marker for ESCC treatment resistance. Utilizing the hypermethylation status of Wnt inhibitors' promoter in plasma as a noninvasive prognostic biomarker for ESCC may prove to be a valuable tool for clinicians and researchers alike in monitoring and assessing disease progression.^237^ In CRC, certain Wnt inhibitor genes that involve the Wnt signaling pathway (SFRP1, SFRP2, SFRP5, DKK2, and WIF1) become hypermethylated in the CpG island of the promoter region during the transition from normal tissue to adenoma. This leads to a decrease in their expression and an increase in the activity of the Wnt pathway within the cell nucleus. The hypermethylation of these genes continues to increase as CRC progresses from adenoma to carcinoma.^238^

In lung cancer, as mentioned earlier, proper lung development and differentiation rely on the precise regulation of the Wnt/β‐catenin signaling pathway. However, an imbalance in this pathway may result in various lung disorders, including cancerous and noncancerous conditions.^239^ Epigenetic mechanisms play a significant role in the development of lung cancer by impacting Wnt pathway genes. Specifically, hypermethylation on the promoter regions of these genes can lead to the silencing or downregulation of their expression. This hypermethylation affects both the ligands/receptors and antagonists/inhibitors of the Wnt pathway, influencing its activity and function in lung cancer cells. Additionally, aberrant methylation of Wnt pathway genes can also disrupt the function of negative regulators within the pathway, further contributing to disease progression.^240^, ^241^ As a result, the Wnt/β‐catenin pathway is considered one of the critical signaling pathways involved in lung cancer. According to the data presented in Table 2, a thorough examination of the genes implicated in the Wnt signaling pathway has revealed that they exhibit abnormal hypermethylation in cases of lung cancer. Based on, NSCLC cell‐line research suggests Wnt signaling activation is caused by the Wnt inhibitor's downregulation, particularly hypermethylation, which contributes to NSCLC dedifferentiation, development, and high stage.^232^ Hypermethylation of Wnt antagonist genes can reduce the expression of these genes and enhance the activation of the Wnt pathway by removing the negative feedback.^240^, ^241^ The level of aberrant methylation of Wnt inhibitors genes increases as lung tumorigenesis advances, which leads to the deactivation of Wnt inhibitors genes as a consequence of hypermethylation in their promoter region.^242^ Based on the evidence in LUAD, abnormal methylation of Wnt antagonists’ genes as epigenetic alterations are detected in precursor lesions.^242^ Approximately 69% of NSCLC cell lines demonstrated elevated active Wnt signaling, which appeared to be associated with the silencing of Wnt antagonist genes (downregulation of Wnt inhibitor genes) caused by promoter hypermethylation.^242^ One of the most well‐known antagonists of the Wnt signaling pathway is APC, which plays a crucial role in its regulation.^135^ The wild type of *APC* is the fundamental component of the Wnt signaling cascade regulation.^133^, ^137^ In cancerous lung tissue, a frequent occurrence of hypermethylation in the APC gene has been observed, as previously discussed. *APC* assumes a central role as the core component of the destruction complex that forms inside the cytoplasm, orchestrating the proteolytic degradation of β‐ catenin.^134^, ^137^ Additionally, *APC*’s activity is impaired in certain cancerous conditions, giving rise to a dysregulated impact of Wnt signaling pathways on the regulation of apoptosis and apoptotic cell death.^135^, ^136^ Notably, experimental knockdown of the APC in chemosensitive human SCLC cells activates the Wnt signaling pathway, ultimately leading to the development of chemotherapy resistance. Furthermore, in vitro experiments with chemoresistant cell lines also exhibit heightened Wnt activity. These findings suggest that the activation of the Wnt signaling pathway may represent a potential mechanism underlying the development of chemoresistance in relapsed SCLC.^243^ Evidence suggests that in LUAD, malignancy impairs all the regulators in the Wnt pathway that play a direct or indirect role.^242^ Due to the significant role of abnormal Wnt signaling activation in NSCLC, there is a critical need to develop negative regulators to effectively counter this condition. Several established inhibitors of the Wnt pathway, such as the DKK and sFRP families, as well as WIF‐1, have demonstrated promising anticancer effects against NSCLC. Thus, these inhibitors have emerged as vital resources in combating NSCLC, and their development and usage can significantly impact the management of this disease. By targeting the aberrant activation of Wnt signaling, these negative regulators have the potential to provide novel therapeutic options for the treatment of NSCLC.^244^

**TABLE 2:** A review of genes involved in the Wnt signaling pathway which abnormally hypermethylated in lung cancer.

Gene names	Function	Cell types	References
*Adenomatous polyposis coli (APC)*	APC as a Wnt antagonist is a part of the β‐catenin destruction complex, which can be improved β‐catenin and each degradation complex affinity.	NSCLC	^77^, ^232^, ^233^
*Axis inhibition protein (AXIN)*	AXIN as a Wnt antagonist is a part of the β‐catenin destruction complex and promotes β‐catenin degradation.	NSCLC	^232^, ^245^
*Wnt inhibitory factor‐1 (WIF1)*	An extracellular antagonist that acts by binding to Wnt ligands and consequently prevents their interaction with receptors.	NSCLC	^246^, ^247^, ^248^
*Secreted frizzled‐related protein (SFRP1)*	SFRP1 is a Wnt antagonist that binds to the frizzle receptors and blocks Wnt protein‐mediated signaling. Aberrant methylation in the SFRP1 gene is associated with lung cancer due to its involvement in tumorigenesis and progression.	NSCLC, (SCC)	^227^, ^229^, ^246^
*Secreted frizzled‐related protein 2 (SFRP2)*	SFRP2 is a Wnt antagonist that binds to the frizzle receptors and leads to the blockade of Wnt protein‐mediated signaling.	NSCLC, (SCC)	^227^, ^229^, ^246^
*Secreted frizzled‐related protein 4 (SFRP4)*	SFRP4 is an endogenous extracellular Wnt antagonist that binds to the frizzle receptors and leads to blocking Wnt protein‐mediated signaling and consequently decreasing β‐catenin. SFRP4 is responsible for arresting the cell cycle, inhibiting proliferation and inducing apoptosis.	NSCLC	^242^, ^244^
*Secreted frizzled‐related protein 5 (SFRP5)*	SFRP5 is a Wnt antagonist that binds to the frizzle receptors and blocks Wnt protein‐mediated signaling.	NSCLC	^229^, ^242^, ^248^
*Runt‐related transcription factor 3 (RUNX3)*	The Wnt antagonist RUNX3 prevents the binding of the TCF4‐β‐catenin complex to target promoters and is responsible for the cell cycle and regulation of apoptosis. In lung adenocarcinoma, RUNX3 promoter hypermethylation is disrupted by the Wnt signaling pathway.	NSCLC, SCLC	^232^, ^249^
*Dickkopf1 (DKK1)*	As a prototype of an extracellular Wnt pathway antagonist, Dkk1 is inhibited by the canonical pathway via binding to the LRP5/LRP6 coreceptor.	NSCLC	^232^, ^250^
*Caudal‐related homeobox 2 (CDX2)*	As a tumor suppressor, CDX2 prevents lung cancer proliferation by suppressing the Wnt signaling pathway.	NSCLC	^251^
*Dishevelled‐associated antagonist of β‐catenin (DACT2)*	In lung cancer cells, DACT2 inhibits the Wnt signaling pathway, preventing cancer proliferation. In addition to degrading DVL through a lysosome‐dependent pathway, DACT2 prevents LEF1 from binding to β‐catenin. As result, hypermethylation of the DACT2 promoter region leads to loss of DACT2 expression and, as a result, leads to increased expression of β‐catenin.	Lung cancer NSCLC	^232^, ^252^
*Empty Spiracles Homeobox 2 (EMX2)*	EMX2 is a suppressor gene in lung carcinogenesis. The expression of EMX2 inhibited the canonical Wnt signaling pathway, suppressing cell proliferation and invasion. In lung cancer, EMX2 is epigenetically silenced via methylation in the promoter, leading to aberrant activation of canonical Wnt signaling.	Lung cancer	^232^, ^253^
*Wingless‐type protein 7a (Wnt7a)*	Wnt7a is involved in noncanonical Wnt signaling and also inhibits tumor growth.	NSCLC	^254^

SCC, squamous cell carcinoma lung cancer; NSCLC, non‐small‐cell lung carcinoma.

OncoMed Pharmaceuticals has developed drugs that target the Wnt signaling pathway, a pathway dysregulated in many human tumors.^234^ There are several types of Wnt signaling pathway negative regulators, which include WIF‐1, sFRP family, DKK family, ncRNAs (miRNAs, lncRNAs, circRNAs), natural compounds, drugs, antibodies, and other categories.^244^ One of these agents, OMP‐18R5 (Vantictumab), a fully humanized antibody targeting at least five different Frizzled receptors, demonstrated antiproliferative effects in various human tumors, including lung, breast, colon (wild‐type *APC* and β‐catenin), and pancreatic cancer, and showed synergistic effects when combined with standard chemotherapy in preclinical studies. The first Phase Ia clinical trial reported promising results for OMP‐18R5, with decreased Wnt pathway gene expression and increased expression of differentiation genes. However, the trial also reported adverse events, including fatigue, vomiting, diarrhea, constipation, nausea, and abdominal pain. OMP‐18R5 is in Phase Ib trials in combination with standard chemotherapy for solid tumors, including breast, lung, and pancreas cancers. In xenografts derived from lung cancer patients, OMP‐18R5 has been shown to be effective in reducing the frequency of tumor‐initiating cells, as well as suppressing tumor growth.^244^ These findings highlight the critical role of the Wnt signaling pathway in cancer progression and suggest that targeting this pathway could be a promising strategy for cancer therapy. Identifying specific Wnt proteins that drive cancer progression and stem cell self‐renewal provides a potential avenue for developing targeted therapies that could improve patient outcomes.

### 2.4 Histone modification aberrations in lung cancer

In eukaryotic cells, nucleosomes form fundamental units comprising genomic DNA ensconced around a core histone octamer, encompassing H2A, H2B, H3, and H4, while histone tails serve as sites for PTMs.^255^, ^256^ Notably, various histone PTMs, such as acetylation, methylation, and phosphorylation, exert regulatory control over nucleosomal dynamics, ultimately influencing the accessibility of transcription factors and RNA polymerase to their target genes. This dynamic modulation is achieved through the alteration of charge density at the interface between DNA and histones.^54^, ^257^ Since DNAm‐based biomarkers can address specific clinical concerns, especially in early diagnosis, staging, prognosis, and therapeutic response prediction. As a result, they are currently universally acknowledged as significant biomarkers in the clinical management of lung cancer.^258^

The activation of gene transcription via histone acetylation is recognized as a pivotal determinant in the pathogenesis of lung cancer.^257^ The identification of specific HDAC inhibitors, such as Vorinostat and Panobinostat, has shown considerable promise in preclinical and clinical trials for NSCLC. However, to fully comprehend and harness their antitumor potential, further investigations are imperative.^257^ Histone methylation is an extensively researched pattern of histone modification that exerts dualistic effects (both promoting and inhibiting) on transcription at distinct gene loci, thereby bestowing a profound and intricate role in the etiology of lung cancer.^259^ The methylation of lysine (K) and arginine (R) residues on histone tails is believed to play a significant role in determining chromatin configurations and biological outcomes.^256^ Notably, certain histone methylation modifiers have been identified in cancers with altered activities, indicating their potential roles as oncogenes or tumor suppressors.^256^ Aberrations in histone methylation modifiers have been closely linked to lung cancer.^33^ Various PTMs, including H3K18ac, H4K12ac, H4R3me2, H3K4me2, and H3K27me3, have been detected in 97 lung cancer patients.^260^ Intriguingly, diminished levels of H3K18ac and H3K27me3 were found to be associated with better survival and prognosis rates in SCC histological cases, while a higher expression of the H3K27me3 mark in NSCLC exhibited a positive correlation with improved prognosis and extended overall survival rates.^261^ Additionally, lower H3K9ac levels were observed in stage I lung‐adenocarcinoma (AD) patients with a better prognosis.^262^ A comprehensive study conducted by Song et al. in 2012, a global status of PTMs on both H3 and H4 for AD and SCC lung malignant histological types (of a total 408 NSCLC cases) was identified. The study found weak nuclear staining for H3K9ac, H3K9me3, and H4K16ac, except for H4K20me3, which was related to tumor recurrence and distant metastasis. Overall, histone acetylation patterns (namely H3K9ac) were found to be correlated with a better prognosis, while histone methylation patterns and/or negative nuclear staining were associated with a poor prognosis.^263^

### 2.5 Noncoding RNA regulation

lncRNAs are RNA transcripts that surpass 200 nucleotides in length and do not encode proteins. In the human genome, only a small fraction (1.5%) is involved in protein‐coding while the rest (98.5%) does not encode proteins.^264^ Initially, lncRNAs were considered to be by‐products of the transcription process, but they are now known to play a crucial role in regulating gene expression in cancer development and progression.^265^ LncRNAs can regulate gene expression at various levels, including epigenetic, transcriptional, translational, and posttranscriptional^266^.^267^ A remarkable aspect of the lncRNA‐mediated regulatory network is their capacity to act as scaffolds, facilitating interactions with diverse signaling molecules and regulatory factors. Depending on their bond partners, lncRNAs exert various regulatory functions, such as gene expression, histone methylation, genomic imprinting, and chromatin modifications.^268^

Moreover, lncRNAs function as cofactors of transcriptional factors, modulating the activity of RNA polymerase II or the transcription machinery.^269^ Additionally, lncRNAs can control posttranscriptional processing and translation of mRNAs, such as capping, splicing, editing, transportation, and stability, through specific complementary interactions with target sequences.^266^ This diverse regulatory capability positions lncRNAs to play pivotal roles in an extensive array of physiological and pathological processes within the body, encompassing not only tumorous conditions but also non‐neoplastic diseases.^266^

#### 2.5.1 Oncogenic ncRNA in lung cancer metastasis‐associated LUAD transcript (MALAT1)

**FIGURE 6:**
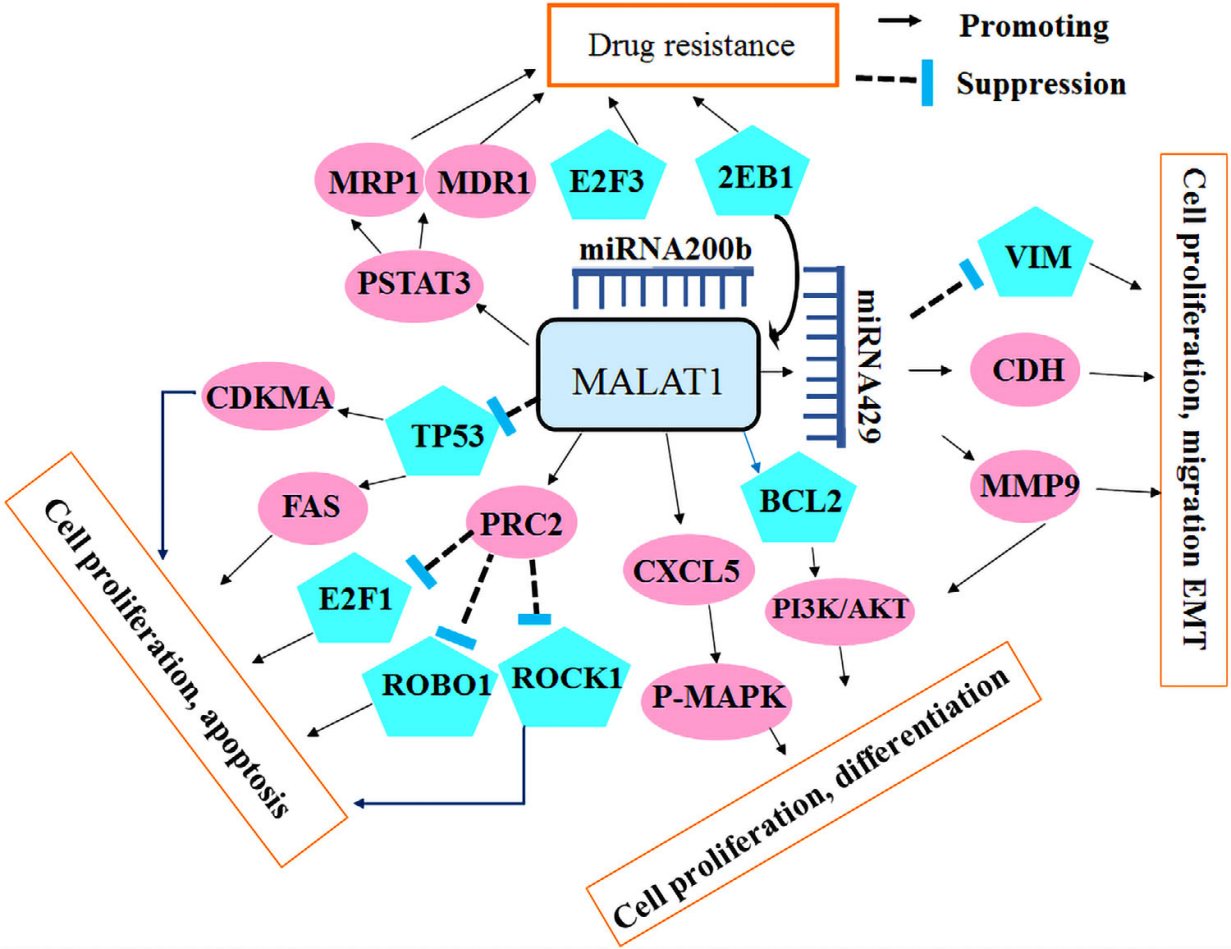
Regulatory network of the long noncoding RNA metastasis associated lung adenocarcinoma transcript 1 (MALAT1) in lung cancer. MALAT1 inhibits the tumor suppressor tumor protein p53 (TP53), thereby regulating the expression of FAS and CDKMA. Additionally, MALAT1 regulates the expression of VIM and matrix metallopeptidase 9 (MMP‐9) through a mechanism mediated by miRNA429. MALAT1 activates the expression of polycomb repressive complex 2 (PRC2), which leads to the inhibition of the expression of E2F transcription factor 1 (E2F1), roundabout homolog 1 (ROBO1), and rho‐associated, coiled‐coil‐containing protein kinase 1 (ROCK1). MALAT1 also upregulates the expression of phospho‐ signal transducer and activator of transcription 3 (STAT3), ATP‐binding cassette sub‐family B member 1 (ABCB1), and multidrug resistance‐associated protein 1 (MRP1). Moreover, MALAT1 acts as a competing endogenous RNA (ceRNA) that regulates the expression of miRNAs, such as miR‐429 and miR‐200b.

MALAT1 is an lncRNA located on chromosome 11q13.1, spanning approximately 8.7 kb in length. It exhibits expression in various human tissues and is conserved across mammals.^270^ Despite its evolutionary conservation, it has been shown to promote cancer cell proliferation, migration, invasion, EMT, and chemoresistance, making it an oncogenic lncRNA.^271^ In the context of NSCLC tissues, MALAT1 expression is notably elevated in tumor tissues compared with adjacent normal tissues, and its overexpression is associated with poor overall survival in NSCLC patients.^272^ Moreover, MALAT1 has been shown to negatively regulate myeloid‐derived suppressor cells (MDSCs) in lung cancer patients.^272^ Silencing MALAT1 in cultured NSCLC cells has been shown to inhibit cell proliferation and colony formation (Figure 6).^273^

##### HOX transcript antisense RNA (HOTAIR)

**FIGURE 7:**
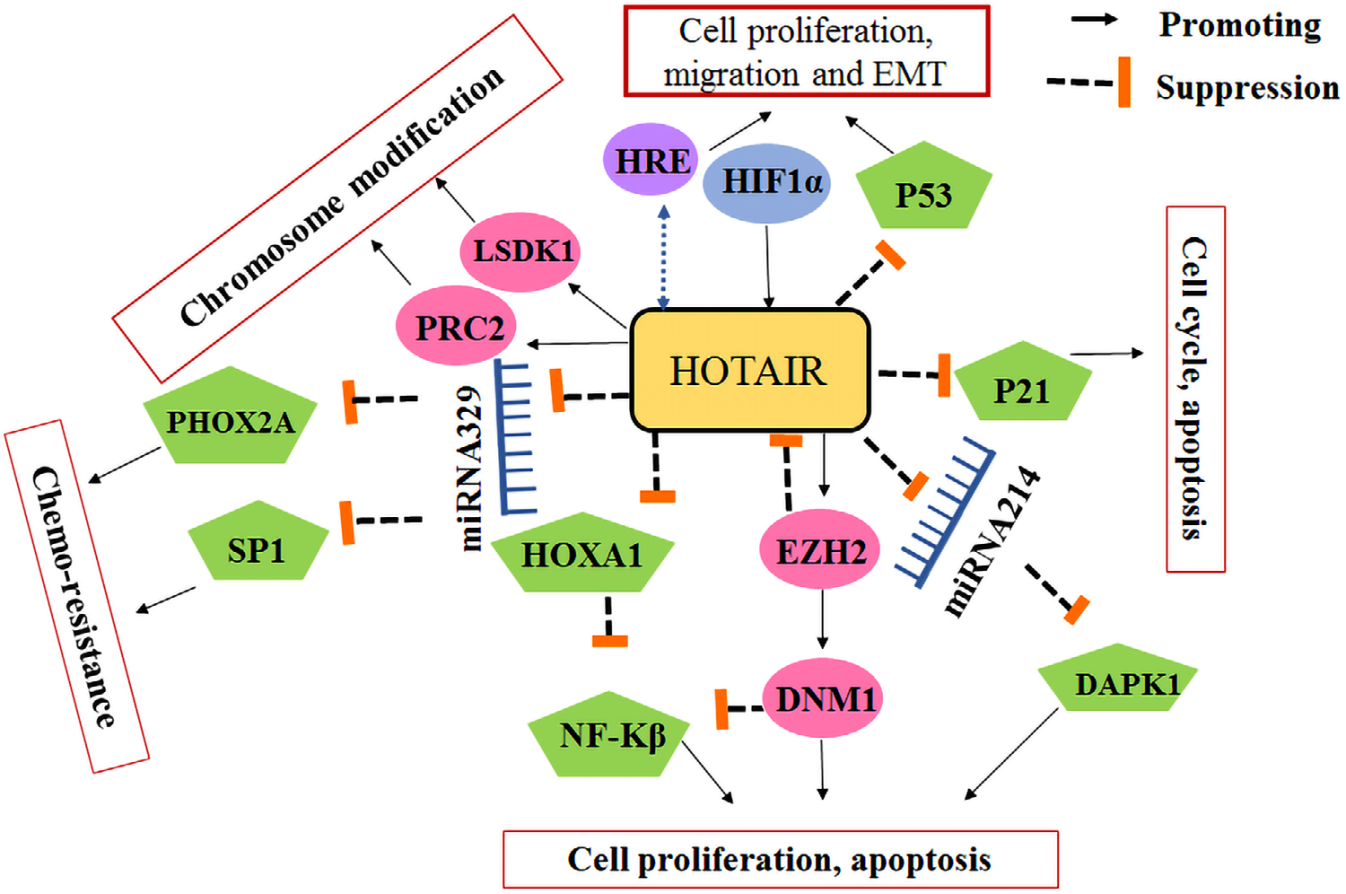
Regulatory network of the long noncoding RNA) HOX Transcript Antisense RNA (HOTAIR)(HOTAIR in lung cancer. HOTAIR regulates the growth and proliferation of lung cancer cells by inhibiting the tumor suppressor p53 and its downstream target p21. Additionally, HOTAIR plays a crucial role in regulating epigenetic gene expression by functioning as a scaffold for polycomb repressive complex 2 (PRC2) and Lysine‐specific demethylase 1 (LSD1), which regulate the expression of genes such as phosphatase and tensin homolog (PTEN), homeobox D10 (HOXD10), β‐catenin, and Wnt inhibitory factor 1 (WIF1). Furthermore, HOTAIR activates the NF‐kB signaling pathway through the methylation of homeobox A1 (HOXA1). HOTAIR can also act as a competing endogenous RNA (ceRNA) to regulate the expression of miRNAs, such as miR‐329 and miR‐214.

HOTAIR is an lncRNA composed of 2.2 kb and 6 exons, located on chromosome 12q13.13 in humans. HOTAIR fulfills a crucial role as a scaffold, intricately orchestrating the assembly of histone modification complexes responsible for the mediation of essential cellular processes, including histone methylation and chromosomal remodeling.^274^ It is transcribed from the antisense strand of the HOXC gene, specifically between HoxC11 and HoxC12, and may regulate gene expression in HOX loci in a cis‐ or trans‐acting manner. The genome encompasses four prominent HOX gene clusters, denoted as HOXA, HOXB, HOXC, and HOXD, collectively encompassing a total of 39 HOX gene family members. The dysregulation of HOTAIR has been detected across multiple cancer types, encompassing lung, pancreatic, breast, colorectal, liver, and gastric cancer.^275^ Particularly in lung cancer, HOTAIR exhibits markedly elevated expression in tumor tissues when compared with adjacent nontumor tissues. Moreover, this aberrant expression is associated with advanced pathological stage, lymph node metastasis, and a dismal prognosis, thus establishing HOTAIR as a negative prognostic factor.^276^ In vitro investigations have revealed the multifaceted role of HOTAIR in regulating apoptosis and the cell cycle, contributing to cisplatin resistance in human LUAD cell (Figure 7).^277^

##### H19

H19 is a maternally expressed lncRNA approximately 2.3 kb in length, situated on chromosome 11p15.5. Comprising five exons and four introns, this evolutionarily conserved lncRNA holds significant relevance in mammalian biology. As the first imprinted gene identified, H19 plays a critical role in embryonic development and tumorigenesis. Moreover, its involvement has been demonstrated in various cancer type, including lung cancer, bladder cancer, ovarian cancer, and pancreatic cancer.^278^ In lung cancer, H19 expression distinctly demonstrates a marked upregulation compared with adjacent normal tissues. Intriguingly, this overexpression extends to the plasma of the patients. Moreover, elevated H19 expression in lung cancer patients correlates significantly with advanced TNM stages, diminished disease‐free survival (DFS), and an unfavorable prognosis. Particularly in NSCLC, H19 actively interacts with miRNA‐p21, fostering cancer progression and exacerbating prognostic outcomes.^279^

MiRNAs are a class of typically composed of approximately 22 nucleotides in length. They can induce translational repression or degradation of target mRNAs by binding to the 30′UTR sequence of the target mRNA, depending on the degree of homology between the miRNA and the target mRNA.^280^ The human genome encompasses an estimated 1,000 transcribed miRNAs, regulating approximately 30% of all genes. As a result, a single miRNA has the capacity to intricately modulate the expression of numerous downstream genes through posttranscriptional processes. The multifaceted functions of miRNAs extend their regulatory reach to various essential biological processes, including cell development, proliferation, differentiation, and apoptosis.^281^ The paramount significance of miRNAs in maintaining normal cellular processes renders their dysregulation a pivotal factor contributing to the onset of various diseases. Intriguingly, numerous studies have identified aberrant miRNA expression patterns in diverse human cancer types, manifesting a complex interplay of both heightened and diminished miRNA levels.^282^ Remarkably, miRNAs exhibit a dualistic role contingent upon their target mRNAs, as they can function either as tumor suppressors or oncogenes.^283^ This intricate regulatory mechanism underscores the paramount importance of miRNAs in cancer biology and unveils promising avenues for therapeutic interventions targeting these versatile molecules.

Numerous investigations have consistently demonstrated the intricate involvement of miRNAs in the realm of epigenetic regulation. Approximately, half of miRNAs associate with CpG islands, making them particularly vulnerable to epigenetic alterations. Emerging evidence from multiple studies highlights the upregulation of some miRNAs following cellular exposure to the agent 5‐aza‐2′‐deoxycytidine and under conditions of DNMT mutations.^284^ In cancer cells, several miRNAs are silenced through CpG hypermethylation, in contrast to their expression in normal cells. For instance, miR‐203 is frequently subjected to DNAm in T cell lymphoma, but not in normal T lymphocytes.^285^ Hypermethylated miRNAs have been identified across various types of cancer, including breast cancer (miR‐9‐1, miR‐124a3, miR‐148, miR‐152, and miR‐663),^286^ colorectal (miR‐124a, miR‐43b, miR‐34c),^284^ oral (miR‐137, miR‐193a),^287^ bladder and prostate tumors (miR‐126),^288^ or HCC (miR‐124, miR‐203, miR‐375).^289^ It is important to note that the epigenetic regulation of miRNA activity is highly specific to the cell and tumor type.

## 3 EPIGENETIC BIOMARKERS FOR LUNG CANCER

Biomarkers represent essential components capable of discerning between normal and aberrant physiological states.^290^ In lung cancer, the imperative for an efficient diagnostic approach to facilitate early detection becomes evident as it holds potential to curtail mortality rates. Cancer biomarkers are specific molecules that can differentiate between normal and cancerous conditions and may aid in the development of more effective diagnostic tools for lung cancer. Biomarkers for cancer can include various biomolecules used for medical purposes, such as proteins, genetic material (DNAs, methylated DNAs, RNAs, and miRNAs), oligosaccharides, lipids, and metabolites. Since cancer is a heterogeneous disease reflecting gene and protein changes within cancer cells, biomarkers can be classified into different categories based on their purpose.

These are the four main purposes or categories of biomarkers in cancer research:

1. *Diagnostic biomarkers*: These biomarkers are used to detect cancer at an early stage or to provide a definitive diagnosis. Notably, elevated levels of specific proteins or genetic markers in blood or tumor samples may serve as indicators of cancer presence. The indispensable significance of these biomarkers lies in their ability to enable early detection and ensure accurate diagnosis of cancer, thereby facilitating timely intervention and improved patient outcomes.
2. *Predictive and staging biomarkers*: These biomarkers assume a crucial role in predicting the likelihood of cancer progression, risk stratification, and staging. Therefore, a biomarker linked to an elevated risk of cancer recurrence or metastasis can provide valuable insights to physicians, aiding them in determining the most appropriate course of treatment. The indispensability of these biomarkers lies in their ability to guide treatment decisions and enhance patient outcomes, emphasizing their pivotal significance in the field of cancer research and clinical practice.
3. *Prognostic biomarkers*: These biomarkers are used to predict the likelihood of cancer recurrence or metastasis, as well as the overall prognosis or outcome of the disease. For example, a biomarker associated with a poor prognosis may indicate the need for more aggressive treatment.
4. *Treatment biomarkers*: These biomarkers are used to guide treatment decisions and predict responses to therapy. For example, a biomarker associated with a specific type of cancer may indicate the need for targeted therapy or immunotherapy. Biomarkers can also be used to monitor treatment response and identify when a treatment is no longer effective.

Overall, identifying and validating biomarkers for early lung cancer detection remains an important area of research to reduce mortality. Biomarker panels utilizing different biomarker categories may provide a promising approach for improving diagnostic accuracy.

### 3.1 MiRNAs as lung cancer biomarkers

MiRNAs have emerged as novel cancer‐specific biomarkers and have been extensively studied for their potential applications in cancer diagnosis and prognosis. MiRNAs regulate gene expression by silencing specific transcripts through base‐pair complementarity. These miRNAs are stably present in plasma and have shown promise in cancer detection. Numerous studies have demonstrated the utility of miRNAs as biomarkers for various types of cancer, including lung, liver, colorectal, stomach, breast, prostate, and cervical cancers.^291^, ^292^ For instance, a study focusing on NSCLC patients' plasma samples identified four isoforms of miRNA (miR‐9, miR‐16, miR‐205, and others) as potential biomarkers for diagnosis and assessing the response to surgical treatment.^293^ Similarly, another investigation identified a subset of miRNAs that could serve as biomarkers for predicting the risk or prognosis of breast cancer development.^294^ Overall, miRNAs show promise as cancer biomarkers due to their stability in plasma and ability to accurately reflect gene expression changes. However, further research and validation studies are necessary to fully exploit their potential in clinical settings for cancer diagnosis, prognosis, and treatment response assessment.

As previously mentioned, miRNAs are short, noncoding RNA molecules that can bind specifically to target mRNA 3′ UTR sequences, resulting in the degradation of the bound mRNA.^295^, ^296^ MiRNAs, as noncoding RNA molecules, possess unique characteristics that enable them to circulate in various body fluids and be internalized by recipient cells through either endocytosis or binding to receptors on the cellular membrane. Therefore, miRNAs in body fluids hold promise as noninvasive biomarkers for diagnosing lung cancer. Numerous studies have confirmed the efficacy of proposed miRNA biomarkers within low‐dose computed tomography lung cancer screening trials, demonstrating their value in predicting the risk of lung cancer in asymptomatic individuals (as summarized in Table 3). These findings strongly support the potential incorporation of miRNA biomarkers into clinical practice, representing a significant advancement in lung cancer screening accuracy and a notable reduction in mortality rates. Among the miRNAs studied extensively in lung cancer, miRNA‐34 stands out as a prominent candidate. Research on miRNA‐34 spans from its initial discovery and validation to exploring its clinical relevance in both diagnosis and therapeutic applications. MiRNA‐34 was initially recognized as one of the p53‐regulated miRNAs, consisting of miR‐34a, miR‐34b, and miR‐34c.^297^ Numerous studies have demonstrated that the miRNA‐34 family members are downregulated in lung cancer, and their restoration has shown the ability to inhibit tumor growth and enhance chemosensitivity. These compelling findings strongly suggest that miRNA‐34 holds tremendous promise as both a valuable biomarker for lung cancer and an attractive therapeutic target.^298^

**TABLE 3:** Selected lung cancer miRNAs biomarkers.

miRNA	Main application‐histological types	Biomarker values	Biofluids tested	References
MiRNA‐34	Early –stage NSCLC	80% accuracy (to heavy smoker high risk) Benning vs. Malignant discrimination	Serum	^298^
Panels of miR‐660, miR‐140‐5p, miR‐451, miR‐28‐3p, miR‐30c, miR‐92a,	NSCLC and SCLC	(For prediction) Sensitivity: 80% Specificity: 90%	Plasma	^299^
Panels of miR‐21, miR‐126, miR‐210, and miR486‐5p	NSCLC	Sensitivity: 86% Plasma (92% for ADC, 82% for SQLC) Specificity: 97% (For stage I sensitivity: 73%, specificity: 97%)	Plasma	^300^

### 3.2 Exosomes

Extracellular vesicles are typically classified into three main categories: macrovesicles, apoptotic bodies, and exosomes.^301^ Exosomes are small nanovesicles, ranging from 30 to 150 nm in size, that were first identified in 1983 in two simultaneous publications.^302^ These vesicles play a crucial role in intercellular communication and are secreted by various cell types, including cancer cells. Recent studies have suggested that exosomes may serve as potential biomarkers for lung cancer diagnosis and monitoring. However, more research is needed to validate their clinical utility and explore their usefulness in combination with other biomarkers for lung cancer detection.

Exosomes are released by every cell in a constitutive manner, but research has shown that tumor cells release higher amounts of exosomes than healthy cells. As a result, exosomes can be found in various body fluids such as blood, semen, and ascites. Exosomes have been found to contain messenger RNA (mRNA), miRNAs, double‐stranded DNA (dsDNA), siRNA, and proteins that could serve as diagnostic, predictive, and prognostic biomarkers for different types of tumors, including lung cancer. Moreover, exosomes are considered horizontal cell communicators between cells, and their contents could reflect the cell of origin, making them a new and accessible source for tumor profiling analysis.^302^

**FIGURE 8:**
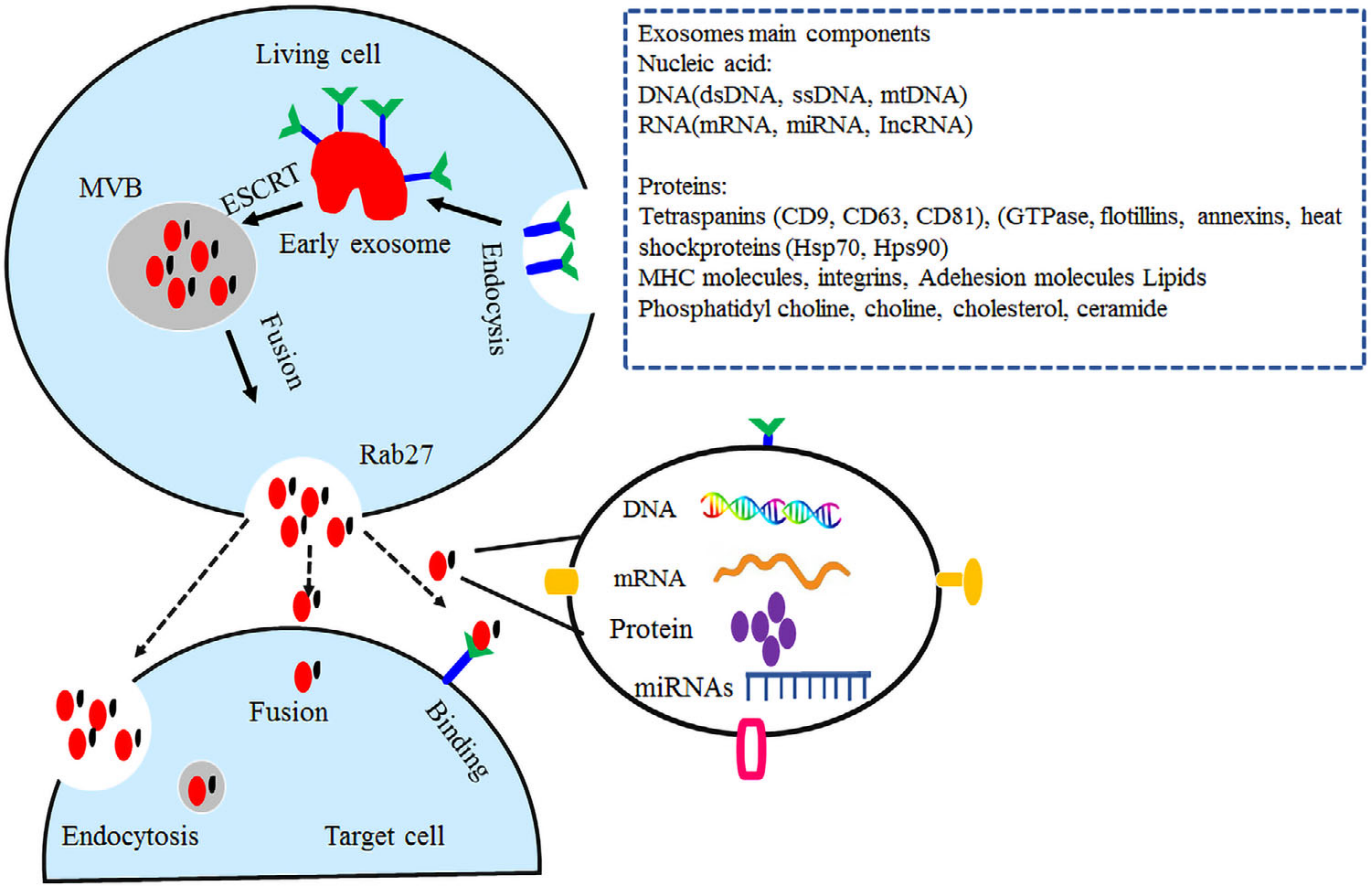
Process of exosome formation and release. This process is highly regulated and begins with the formation of early endosomes in the cell membrane. These early endosomes mature into multivesicular bodies (MVBs), which contain the exosomes. The MVBs then fuse with the cell membrane and release the exosomes into the extracellular space under the regulation of ras‐related protein Rab‐27A (Rab27). Exosomes can enter target cells through three different mechanisms: fusion, endocytosis, and protein–receptor interactions. Understanding the complex process of exosome formation and release is important for comprehending their role in cellular communication and disease pathogenesis. A visual representation of this process can be found in the provided figure which is important for understanding their role in cellular communication and disease pathogenesis.

Statistical data have shown that, exosomes contain a significant amount of proteins, mRNAs, miRNAs, and lipids, with 9769 proteins, 3408 mRNAs, 2838 miRNAs, and 1116 lipids being identified. The proteins found in exosomes are responsible for their structure, biosynthesis, and fusion. For instance, the exosome's surface is composed of a lipid bilayer and specific proteins, including tetraspanins (CD9, CD63, CD81, and CD82), receptors, and integrins, which are unique to different cells. This surface molecule specificity enables the exosome to target specific cells. In addition to containing proteins responsible for their structure, biosynthesis, and fusion, exosomes also contain heat shock proteins, such as HSP60 and HSP90, which ensure proper protein folding and stress response.^303^ Other proteins found in exosomes include ALIX and TSG101, which are involved in multivesicular body (MVB) production,^304^ as well as membrane transport and fusion‐related proteins, such as the RAB family, annexins, and flotillins. A visual representation of exosome formation and context can be found in Figure 8.^305^

The exosomal markers CD151, CD171, and tetraspanin 8 have been identified as the most significant differentiators among patients with lung cancer, regardless of the stage or histological subtype.^306^ Exosomes play a crucial role in cell‐to‐cell communication and have a significant impact on various stages of the metastatic process. Specifically, exosomes derived from lung cancer cells promote cell proliferation, angiogenesis, and metastasis, as well as regulate drug resistance and antitumor immune responses during lung carcinogenesis. These exosomes are currently being investigated as an essential component in liquid biopsy assessments for lung cancer diagnosis.^306^, ^307^ Furthermore, the exploration of exosomal components, such as RNAs and proteins, as potential diagnostic and prognostic biomarkers, shows promising results.^306^ Previous studies have demonstrated that exosomal molecules derived from cancer cells and liquid biopsies can serve as biomarkers for cancer diagnosis and prognosis. Therefore, exosomes hold significant potential as diagnostic and predictive biomarkers in the context of lung cancer.^308^

#### 3.2.1 Exosomes are involved in lung cancer proliferation

Cell proliferation is a fundamental process in human growth, development, and reproduction, characterized by changes in the expression or activity of proteins related to the cell cycle. These changes are known to play a crucial role in the development of lung cancer.^309^ For instance, a study conducted by Janowska‐Wieczorek et al.^310^ revealed that exosomes derived from platelets transferred glycoprotein IIbIIIa (CD41) to the surface of lung cancer cells, leading to the upregulation of cyclinD2 expression and promoting the phosphorylation of MAPKp4244. Furthermore, the exosomes mentioned above were found to induce the proliferation of lung cancer cells, highlighting the role of exosomes in regulating lung cancer cell proliferation. These findings suggest the potential use of exosomes as targets for cancer therapy in the future.^310^ KLF9, a member of the KLF family, is known to regulate cell proliferation. Research has shown that miR‐660‐5p levels are significantly higher in the plasma of patients with NSCLC. Furthermore, miR‐660‐5p promotes NSCLC progression by targeting KLF9, suggesting a potential role in the regulation of cell proliferation in lung cancer. Exosomes have also been found to play a significant role in different types of cancer, including lung cancer. For instance, a study has shown that exosomes containing miR‐96 from H1299 cells, a human LUAD cell line, promote cell proliferation by targeting and inhibiting LMO7. These findings suggest that miR‐96 may participate in the promotion of cell proliferation in lung cancer through exosomes.^311^ In addition, a study by Fabbri et al.^312^ found that miR‐29a and miR‐21 are significantly upregulated in exosomes isolated from A549 cells, a human LUAD cell line. The upregulation of these miRNAs occurs when they bind to toll‐like receptors in surrounding immune cells and has been linked to the proliferation and metastasis of lung cancer cells. These findings suggest that exosomes play a crucial role in the communication between cancer cells and the immune system, which could provide new targets for cancer therapy.^312^ Understanding the mechanisms underlying exosome‐mediated tumor progression in lung cancer is important for the development of novel therapeutic strategies that target exosomes and their contents. Further research is necessary to fully elucidate the complex processes involved in exosome‐mediated communication and their potential use in lung cancer treatment.

#### 3.2.2 Exosomes play a critical role in EMT and the metastasis of lung cancer

**FIGURE 9:**
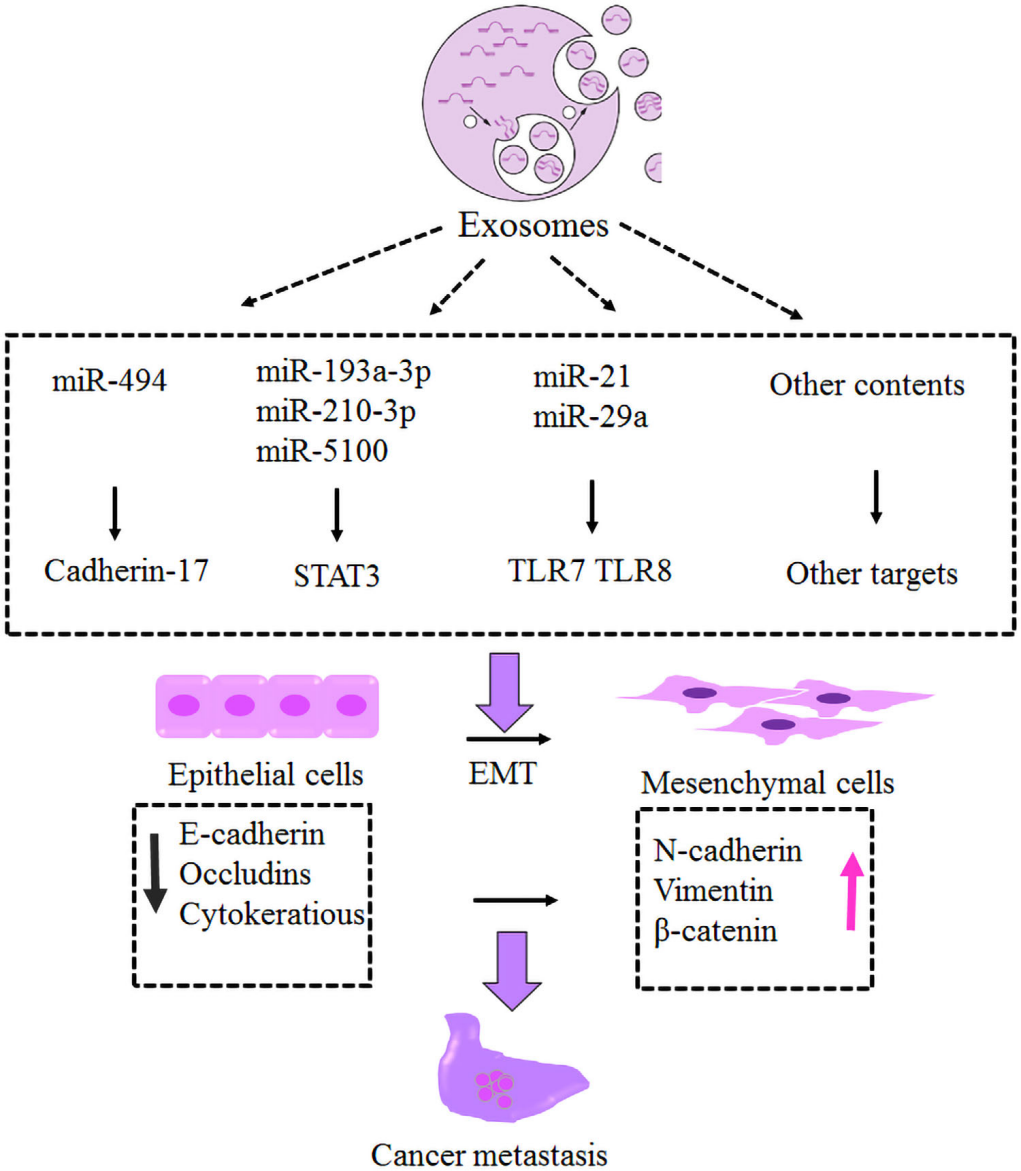
Exosomes play an integral role in epithelial–mesenchymal transition (EMT) and tumor metastasis. These exosomes can influence multiple signaling pathways involved in EMT, leading to the loss of epithelial markers such as E‐cadherin and cytokeratins, and the increase of mesenchymal markers such as N‐cadherin and vimentin. This results in the acquisition of the mesenchymal phenotype, which is associated with increased invasiveness and promotes tumor metastasis. Understanding the role of exosomes in EMT regulation is important for the development of new therapeutic strategies to target this complex process and prevent tumor metastasis.

EMT is a complex process that involves the transformation of polarized epithelial cells, which are connected via adhesions, into mesenchymal cells with migratory and invasive properties. During EMT, epithelial cells lose their cell polarity and adhesion properties and acquire motility properties, which enable them to migrate and invade surrounding tissues. EMT is accompanied by changes in gene expression, cytoskeletal organization, and extracellular matrix composition, which collectively lead to the acquisition of mesenchymal characteristics. EMT is involved in various physiological and pathological processes, including embryonic development, wound healing, and cancer metastasis. Understanding the mechanisms that regulate EMT is crucial for developing new therapeutic strategies for diseases such as cancer.^313^ During the EMT process, the expression of E‐cadherin is either decreased or absent between cells, whereas N‐cadherin and vimentin are overexpressed. This process can induce the mesenchymal phenotype, which is associated with highly aggressive tumor cells, promoting tumor proliferation and metastasis in cancer progression. Several studies have emphasized the significant roles of exosomes in EMT, as depicted in Figure 9. Exosomes can transport specific miRNAs, growth factors, cytokines, and extracellular matrix components that can modulate the EMT process and contribute to tumor progression.^314^ As an example, Tang et al.^315^ conducted a study that examined the changes in miRNA content in exosomes from human NSCLC cell lines (A549 and H1299 cells) that underwent EMT. The findings of this study showed that certain exosomal miRNAs derived from intercellular phenotype cells were linked to the progression of EMT. This suggests that exosomes play an important role in the regulation of EMT‐related processes and may be involved in the development and progression of cancer.^315^ Another study conducted by Rahman et al.^316^ investigated the effect of exosomes on the EMT process in lung cancer. The study found that exosomes derived from the serum of lung cancer patients and from highly metastatic human lung cancer cell lines (PC14HM, LUAD) were capable of inducing the EMT process in human bronchial epithelial cells. This, in turn, significantly increased the proliferation and invasion of lung cancer. These findings suggest that exosomes play a crucial role in the regulation of EMT and may contribute to the progression of lung cancer.^316^ Indeed, studies have reported that exosomes derived from metastatic or advanced lung cancer cells can induce the expression of vimentin and promote EMT in human bronchial epithelial cells.^316^ This process could lead to the alteration of cancer cell adhesion through the modulation of the VAV2‐Rac1 pathway and the modification of focal adhesion kinase activity in lung cancer.^317^ Exosomes can transfer specific miRNAs from stromal cells to epithelial cells, which can modulate the expression of genes involved in cancer progression. Additionally, bone marrow‐derived mesenchymal stem cells (BMSCs) are a type of multipotent stromal cell that is present in the lung cancer microenvironment.^318^ Studies have shown that BMSCs play a crucial role in promoting cancer metastasis through various mechanisms, including the induction of EMT, the promotion of angiogenesis, and the modulation of the immune system. Moreover, BMSCs have been shown to secrete exosomes that can modulate the TME and promote cancer progression. These findings suggest that BMSCs and their exosomes could be potential therapeutic targets for the treatment of lung cancer.^319^ Further research is necessary to fully elucidate the complex mechanisms involved in BMSC‐mediated cancer progression and their potential use in lung cancer treatment.

## 4 THERAPEUTIC INTERVENTION TARGETING EPIGENETIC CHANGES

Currently, standard treatments for most patients with lung cancer include surgery, chemotherapy, radiation therapy, targeted drugs, or a combination of these treatments.^320^ Targeted drugs can work differently from chemotherapy drugs. Sometimes, when chemotherapy drugs are not more effective, they can work very well and also do not have the side effects of methods like chemotherapy. Targeted drugs can be used independently or together with chemotherapy.^321^ In this section, examples of these drugs are given:

### 4.1 Drug targets

Recent advancements in our understanding of the molecular mechanisms underlying NSCLC have led to the development of several targeted drugs for the treatment of this disease. Targeted drugs are designed to specifically target and inhibit the activity of molecules that are overexpressed or mutated in cancer cells. By targeting these molecules, these drugs can halt the growth and spread of cancer cells while minimizing harm to healthy cells. In NSCLC, several molecular targets have been identified, including epidermal growth factor receptor (EGFR), vascular endothelial growth factor (VEGF), V‐RAF mouse sarcoma virus oncogene homolog B1 (BRAF), mesenchymal–epithelial transition factor (MET), rearranged in transfection (RET), receptor tyrosine kinase ROS proto‐oncogene 1 (ROS1), Kirsten rat sarcoma virus (KRAS), anaplastic lymphoma kinase (ALK), human epidermal growth factor receptor 2 (HER2), neurotrophic tyrosine receptor kinase (NTRK), and phosphoinositide 3‐kinase (PI3K) can change expression as a result of mutation. These factors can act as oncogenic factors in lung cancer.^322^ The drugs investigated in this study target EGFR, VEGF, KRAS, ALK, ROS1, MET, NTRK, BAF, RET, HER2, and PI3K, which are described below.

#### 4.1.1 Drugs that target EGFR

The EGFR is a protein that is found on the surface of cells and is involved in the regulation of cell growth and division. When EGFR binds to specific ligands, such as epidermal growth factor (EGF) or transforming growth factor alpha (TGF‐alpha), it triggers a series of intracellular signaling pathways, including the PI3K/Akt and MAPK pathways. These pathways are involved in regulating various cellular processes, such as cell proliferation, differentiation, migration, and apoptosis (cell death).^323^ Abnormal activation of the EGFR pathway, through mutations or overexpression of EGFR or its ligands, has been implicated in the development and progression of various types of cancer, including NSCLC, making EGFR an important target for cancer treatment. Since EGFR is overexpressed in more than 60% of NSCLCs, it is a critical target for the treatment of these tumors. The most common mutations in EGFR found in NSCLC are exon 19 deletion and exon 21 (L858R) substitution mutations. These mutations lead to the constitutive activation of the EGFR signaling pathway, which promotes cell proliferation, survival, and angiogenesis, driving tumor growth and progression.^323^

EGFR inhibitors can reduce the growth of tumor cells through disruption of the signaling pathway (Ras). Argeted drugs such as erlotinib (Tarceva), afatinib (Gilotrif), gefitinib (Iressa), osimertinib (Tagrisso), and dacomitinib (Vizimpro), which inhibit EGFR tyrosine kinase activity, have been developed and approved for the treatment of NSCLC harboring EGFR mutations. These drugs have shown significant clinical benefits in terms of tumor response rate, progression‐free survival, and overall survival in patients with EGFR‐mutated NSCLC. These drugs can be used independently as the main treatment for NSCLC. According to studies, erlotinib is sometimes used in combination with drugs that inhibit the angiogenesis pathway. Moreover, osimertinib can be used as a combined treatment after surgery.^324^

Erlotinib and gefitinib are the first‐generation drugs in this group, and their action is reversible. Afatinib and dacomitinib are the second‐generation drugs in this group that covalently bind to EGFR, resulting, in irreversible inhibition. Osimertinib is considered the third‐generation drug in this group, and it is preferred over other drugs in metastatic NSCLC patients.^325^

Erlotinib reversibly binds to the adenosine triphosphate (ATP) binding site of the EGFR and inhibits its phosphorylation. This drug is used to treat metastatic NSCLC caused by specific EGFR mutations, including exon 19 deletions or exon 21 (L858R) substitutions. The safety and efficacy of erlotinib in treating NSCLC patients with EGFR mutations other than exon 19 deletions or exon 21 (L858R) substitutions have not been established. On the other hand, gefitinib works similarly to erlotinib by selectively targeting the mutated EGFR protein in tumor cells.^326^, ^327^

Afatinib is a tyrosine kinase inhibitor (TKI) that belongs to the 4‐anilinoquinazoline class of drugs and is formulated as a di‐alkyl maleate salt. This drug selectively blocks the ErbB family by covalently binding to all homo‐ and heterodimers formed by EGFR (ErbB1), HER2 (ErbB2), ErbB3, and ErbB4 family members and irreversibly blocks signaling through inhibition of phosphorylation.^328^, ^329^

Dacomitinib, also known as (2E)‐N‐16‐4‐(piperidin‐1‐yl) but‐2‐enamide, is an orally administered, highly selective, second‐generation TKI. This drug exerts its inhibitory effect by irreversibly binding to ATP through a quinazoline scaffold. By binding to ATP, dacomitinib inhibits the activity of specific tyrosine kinases, which can slow or stop the growth of cancer cells.

Osimertinib is approved for the treatment of metastatic NSCLC caused by the T790M mutation in EGFR. This mutation is associated with poor prognosis in late‐stage disease due to increased ATP binding activity to EGFR. Many NSCLC patients become resistant to first‐generation EGFR inhibitors over time, and osimertinib has been shown to be effective in this group. One advantage of osimertinib over second‐generation drugs is its lower toxicity profile, while still maintaining high efficacy.^330^, ^331^

#### 4.1.2 Drugs that target VEGF

Tumors grow through a process called angiogenesis, which involves the formation of new blood vessels to supply nutrients and oxygen to the tumor. VEGF is a key regulator of angiogenesis and has proangiogenic activity that plays a significant role in both healthy and unhealthy angiogenesis processes. There is ample evidence to suggest that VEGF is critical for the survival and proliferation of cancer cells and plays an essential role in the angiogenesis process, including lymphangiogenesis in tumor cells.^332^

Bevacizumab and ramucirumab are monoclonal antibodies (IgG antibodies) that specifically target VEGF, a protein that plays a crucial role in angiogenesis. These IgG antibodies can be used in combination with chemotherapy as an initial treatment for advanced NSCLC or as a standalone treatment after chemotherapy. Both bevacizumab and ramucirumab are IgG antibodies, which are a type of antibody that can be produced in large quantities and have a longer half‐life in the body than other antibody types. Monoclonal antibodies such as bevacizumab and ramucirumab bind to VEGF and prevent its interaction with cell surface receptors, thereby inhibiting receptor phosphorylation. This leads to the inhibition of tumor cell proliferation, permeability, and migration. These drugs also prevent the formation of new blood vessels, which reduces the blood supply to tumor cells and limits their growth.^333^ Research suggests that patients with COVID‐19 may experience overexpression of VEGF, which can worsen lung pathology, including acute respiratory distress syndrome and acute lung injury. Given the potential role of VEGF in COVID‐19‐related pulmonary complications, the effects of bevacizumab are being investigated as a potential treatment option for severe cases of COVID‐19.^334^ However, more research is needed to determine the safety and efficacy of bevacizumab for treating COVID‐19.

#### 4.1.3 Drugs that target KRAS

KRAS mutations, particularly the G12C mutation, occur in about 13% of NSCLCs and can cause abnormal protein expression, leading to the growth and expansion of tumor cells. The KRAS gene is one of the oncogenic factors in NSCLC. Drugs such as sotorasib (Lumakras) and adagrasib (Krazati) can specifically target the KRAS G12C protein and prevent the growth and spread of tumors in NSCLC.^335^, ^336^ Sotorasib has been approved by the United States Food and Drug Administration (US FDA) for the treatment of advanced NSCLC with KRAS G12C mutations, while adagrasib is still in clinical trials. In 2021, the US FDA approved sotorasib as a treatment for advanced and metastatic NSCLC with KRAS G12C mutations. Sotorasib is a polyacrylamide derivative that works by irreversibly binding to the P2 subunit of the inactive GDP‐bound KRAS, deactivating the KRAS protein by establishing a covalent bond with the cysteine residue at position 12 of the KRAS G12C mutant. Typically, the KRAS protein is activated when GTP binds to it, which activates the MAP kinase pathway. By inhibiting KRAS signaling, sotorasib can lead to the cessation of tumor growth and induce apoptosis. Sotorasib is a small molecule inhibitor of the RAS GTPase family, which includes KRAS, and is the first drug to target KRAS directly.^337^, ^338^, ^339^

Adagrasib is the second drug approved by the US FDA for the treatment of NSCLC with KRAS G12C mutations. Similar to sotorasib, adagrasib also binds to the cysteine residue of KRAS G12C mutations. Substitution of cysteine with glycine at position 12 in KRAS (KRASG12C) impairs GTP hydrolysis, causing KRAS to remain in its active form. The use of adagrasib results in the binding of GDP to KRAS and the irreversible deactivation of this protein.^340^, ^341^

#### 4.1.4 Drugs that target ALK

Rearrangements in the ALK gene can result in the abnormal production of the ALK protein, leading to increased growth and spread of cancer cells. In NSCLC, ALK fusions involving EML4, KIF5B, KLC1, and TPR have been identified.^342^ These gene fusions can lead to the expression of a fusion protein that has constitutive kinase activity, promoting tumor growth and progression. Targeted therapies, such as ALK inhibitors, have been developed to specifically target the ALK fusion protein and inhibit its activity, which can slow or stop the growth of cancer cells.

Crizotinib (Xalkori), Ceritinib (Zykadia), alectinib (Alecensa), brigatinib (Alunbrig), lorlatinib (Lorbrena), entrectinib, and ensartinib are among the drugs that inhibit the abnormal ALK protein. These drugs can be effective when tumor cells become resistant to chemotherapy, and they can often shrink tumors in people with advanced lung cancer. Crizotinib was the first drug approved in the group of ALK inhibitors. However, this drug caused many side effects and resistance due to the creation of unfavorable hydrogen bonds with other elements.^343^

Alectinib is an ALK inhibitor that is used in the treatment of NSCLC with ALK gene fusions. By inhibiting ALK, alectinib prevents the phosphorylation and downstream activation of STAT3 and AKT, which are signaling proteins involved in cell growth and survival. This inhibition can lead to decreased tumor cell viability and slow or stop the growth of cancer cells. Alectinib is a targeted therapy that specifically addresses the underlying genetic abnormalities in ALK‐positive NSCLC and has shown promising results in clinical trials.^344^

Brigatinib is a reversible dual inhibitor of ALK and EGFR that has been shown to inhibit other kinases, such as ROS1. Brigatinib is indicated for the treatment of patients with ALK‐positive NSCLC who are intolerant to crizotinib. Similar to alectinib, the mechanism of action of brigatinib involves the inhibition of ALK activity, which prevents the downstream activation of STAT3 and AKT, leading to decreased tumor cell viability. Brigatinib specifically addresses the underlying genetic abnormalities in ALK‐positive NSCLC.^345^, ^346^

Entrectinib is a multikinase inhibitor that has a wider range of targets compared with other ALK inhibitors, such as ceritinib, alectinib, and lorlatinib. In addition to ALK, entrectinib also targets ROS1 and NTRK gene fusions, which are found in a variety of solid tumors, including NSCLC. This wider range of targets makes entrectinib a beneficial treatment option for patients with NSCLC who have ALK, ROS1, or NTRK gene fusions. Entrectinib works by inhibiting the activity of these fusion proteins, which can lead to decreased tumor cell growth and increased cell death. Entrectinib has shown promising results in clinical trials and has been approved by the US FDA for the treatment of NSCLC with ALK, ROS1, or NTRK gene fusions.^347^

Lorlatinib belongs to the third generation of ALK inhibitors, and its safety and efficacy have improved compared with the second generation. Lorlatinib is used when cancer cells become resistant to drugs such as ceritinib, alectinib, crizotinib, or other ALK inhibitors. The antitumor effect of lorlatinib in in vivo models appears to be dose‐dependent and involves the inhibition of ALK phosphorylation. Additionally, lorlatinib has the ability to cross the blood–brain barrier, making it a promising treatment option for patients with brain metastases. Lorlatinib has been shown to partially treat progressive or worsening brain metastases, which is a common complication in patients with ALK‐positive NSCLC.^348^, ^349^

#### 4.1.5 Drugs that target the receptor tyrosine kinase ROS1

In rare cases, rearrangements may occur in the ROS1 gene, and in this subset, the ALK, KRAS, and EGFR genes are usually healthy. Drugs that inhibit ROS1 include ceritinib (Zykadia), crizotinib (Xalkori), lorlatinib (Lorbrena), and entrectinib (Rozlytrek). The mechanism of action of drugs that inhibit ROS1 is similar to that of ALK inhibitor drugs, and they can cause tumor cells to shrink. Crizotinib, lorlatinib, ceritinib, and entrectinib, which are ALK inhibitors, also have the ability to inhibit ROS1, as studies have shown. Crizotinib and ceritinib are suitable drugs for first‐line treatment, and after the tumor becomes resistant to these drugs, chemotherapy is used in later stages. Studies have also shown that entrectinib can be effective in people with metastatic NSCLC who have changes in ROS1 expression.^350^

#### 4.1.6 Drugs that target V‐RAF mouse sarcoma virus oncogene BRAF

Mutations in the BRAF gene activate the RAS–RAF–MEK–ERK pathway, which leads to the proliferation and survival of NSCLC tumor cells. Dabrafenib (Tafinlar) and trametinib (Mekinist) are protein inhibitors of BRAF and MEK, respectively. Dabrafenib directly binds to the BRAF protein, while trametinib inhibits the MEK protein and can prevent the progression of metastatic NSCLC caused by the BRAF (V600E) mutation. Trametinib is currently approved for the treatment of BRAF‐mutated cancers, such as NSCLC, as monotherapy or in combination with dabrafenib to improve therapeutic efficacy. Dabrafenib is a competitive and selective inhibitor of BRAF that binds to the ATP‐binding site. On the other hand, trametinib is a reversible, highly selective, and allosteric inhibitor of MEK that prevents its phosphorylation and exerts its therapeutic effect.^351^, ^352^

#### 4.1.7 Drugs that target RET

A small percentage of NSCLC cases have specific changes in the RET gene that cause abnormal production of the RET protein, leading to increased tumor cell growth. Selpercatinib (Retevmo) and pralsetinib (Gavreto) are RET protein inhibitors that can be used in advanced NSCLC cases with REt alterations. Selpercatinib and pralsetinib are the first generation of specific RET inhibitors used to treat cancers caused by RET mutations. These drugs bind to RET in competition with ATP molecules and inhibit autophosphorylation. Selpercatinib and pralsetinib are believed to have a better safety profile than other multikinase inhibitors because they are specific RET inhibitors. The difference between these two drugs is their side effects.^353^, ^354^, ^355^

#### 4.1.8 Drugs that target MET

The MET is a tyrosine kinase receptor that undergoes changes in expression in many tumors, making it a potential target for gene therapy. One class of targeted drugs that has been approved for the treatment of NSCLC is TKIs. TKIs are drugs that inhibit specific proteins involved in cell signaling pathways, such as the EGFR and the anaplastic lymphoma kinase (ALK). Mutations in these genes are common in NSCLC and can drive tumor growth. TKIs can help block these signaling pathways and slow down or stop tumor growth. Currently, at least seven TKIs targeting MET mutations are available commercially or in clinical trials. Capmatinib (Tabrecta), tepotinib (Tepmetko), (Cabometyx), cabozantinib, glesatinib, and merestinib are MET inhibitors that directly target the MET protein and reduce the growth of tumor cells in NSCLC. Additionally, crizotinib, which inhibits ALK and ROS1, can also have an inhibitory effect on MET.^356^, ^357^

Capmatinib is a kinase inhibitor targeting c‐Met receptor tyrosine kinase in the treatment of NSCLC caused by MET exon 14 skipping. Aberrant c‐Met activation has been observed in many cancers, including NSCLC. Aberrant expression of c‐Met leads to the over activation of multiple downstream signaling pathways such as STAT3, PI3K/ATK, and RAS/MAPK. Capmatinib inhibits c‐Met phosphorylation and thus affects NSCLC.^358^, ^359^

Tepotinib inhibits the phosphorylation of MET and subsequent signaling pathways, leading to the inhibition of growth and migration of tumor cells. This drug also helps in tumor suppression by regulating the expression of the EMT suppressor.^360^

Glesatinib is another drug that is being researched for the treatment of NSCLC. Studies on tumor models have shown that glesatinib can have an inhibitory effect on the MET and can reduce metastasis after resistance to drugs such as crizotinib.^361^

Merestinib was originally developed to target MET and is an oral kinase inhibitor with antitumor, antiangiogenic, and antiproliferative activity in tumor models. Further studies have shown that merestinib is a multitarget inhibitor and has the ability to inhibit tyrosine kinases such as ROS1 and NTRK. However, this drug is currently in the research phase and is not yet approved for clinical use.^362^

#### 4.1.9 Drugs that target HER2

Although the HER2 gene is one of the most important biomarkers in breast cancer, a small percentage of NSCLC cases can have changes in the HER2 gene.

Trastuzumab deruxtecan (Enhertu) is a combination of an antibody and a chemotherapy drug. This drug is an antibody against HER2 that is conjugated to a topoisomerase inhibitor (deruxtecan). After Trastuzumab deruxtecan binds to HER2 in cancer cells, the bond between the antibody and deruxtecan is broken due to the activity of lysosomal enzymes. Next, deruxtecan passes through the cell membrane and induces apoptosis in tumor cells through DNA damage. This drug is used in the treatment of inoperable metastatic NSCLC that expresses HER2.^363^

#### 4.1.10 Drugs that target NTRK

A rare number of NSCLCs may have alterations in one of the NTRK genes. Larotrectinib (Vitrakvi) and entrectinib (Rozlytrek) are drugs that inhibit the abnormal growth of NSCLC tumor cells by inactivating the proteins produced by the NTRK gene. Larotrectinib inhibits the activity of NTRK by binding to a neurotrophin, inhibiting growth, and inducing apoptosis in lung cancer cells. On the other hand, entrectinib can act as an ATP competitor and is an inhibitor for all three NTRK, ALK, and ROS1 factors, making it useful for a wide range of applications.^364^, ^365^

#### 4.1.11 Drugs that target PI3K mutations

The PI3K signaling pathway regulates various cellular processes, including cell proliferation, differentiation, and apoptosis, as well as gene transcription and protein synthesis.^365^, ^366^

Studies have shown that in rare cases of NSCLC, mutations that occur in the PIK3CA and PI3K/AKT/mTOR pathways can be inhibited by increasing PTEN protein expression.^367^ LY294002 is a PI3K inhibitor that is currently in the laboratory phase.

Studies show that several miRs can reverse the inhibition of tumor growth, progression, and metastasis caused by the PI3K/AKT/mTOR pathway. Furthermore, miRs can regulate tumor resistance in NSCLC by targeting the PI3K/AKT/mTOR pathway. For instance, miR‐328 inhibitors can improve cisplatin sensitivity in NSCLC cells by regulating PTEN expression. Moreover, miR‐126, miR‐203, and miR‐34a have also been shown to regulate drug resistance through PI3K/AKT signaling.^368^, ^369^

Table 4 displays the list of these drugs along with their type, brand name, drug form, chemical formula, and US FDA approval status for NSCLC. It should be noted that some of these drugs have been approved by the US FDA for the treatment of other cancers.

**TABLE 4:** Target drug for treatment of non‐small cell lung cancer (NSCLC).^1^

Generic name	Brand name	Target	Drug forms	Type	Chemical formula	Status^2^
Erlotinib	Tarceva	EGFR	Tablet	Small molecule	C22H23N3O4	2016Approved
Gefitinib	Iressa	EGFR	Tablet	Small molecule	C22H24ClFN4O3	2015 Approved
Afatinib	Gilotrif	EGFR	Tablet	Small molecule	C24H25ClFN5O3	2013 Approved
Dacomitinib	Vizimpro	EGFR	Tablet	Small molecule	C24H25ClFN5O2	2018 Approved
Osimertinib	Tagrisso	EGFR	Tablet	Small molecule	C28H33N7O2	2020 Approved
Necitumumab	Portrazza	EGFR	Injection	Biotech (protein‐based therapies)		2015 Approved
Bevacizumab	Avastin	VEGF	Injection	Biotech (protein‐based therapies)	C6538H10034N1716O2033S44	2006Approved
Ramucirumab	Cyramza	VEGF	injection	Biotech (protein‐based therapies)	C6374H9864N1692O1996S46	2020 Approved
Sotorasib	Lumakras	KRAS	Tablet	Small molecule	C30H30F2N6O3	2021 Approved
Adagrasib	Krazati	KRAS	Tablet	Small molecule	C32H35ClFN7O2	2022 Approved
Crizotinib	Xalkori	ALK, ROS1, MET	Capsule	Small molecule	C21H22Cl2FN5O	2016 Approved
Ceritinib	Zykadia	ALK, ROS1	Capsule, Tablet	Small molecule	C28H36ClN5O3S	2014 Approved
Alectinib	Alecensa	ALK	Capsule	Small molecule	C30H34N4O2	2015Approved
Brigatinib	Alunbrig	ALK, EGFR	Tablet	Small molecule	C29H39ClN7O2P	2020 Approved
Lorlatinib	Lorbrena	ALK, ROS1	Tablet	Small molecule	C21H19FN6O2	2021 Approved
Ensartinib		ALK	Capsule	Small molecule	C26H27Cl2FN6O3	Investigational
Entrectinib	Rozlytrek	ALK, ROS1, NTRK	Capsule	Small molecule	C31H34F2N6O2	2019 Approved
Dabrafenib	Tafinlar	BRAF	Capsule	Small molecule	C23H20F3N5O2S2	2023 Approved
Trametinib	Mekinist	BRAF	Tablet, solution	Small molecule	C26H23FIN5O4	2017Approved
Selpercatinib	Retevmo	RET	Capsule	Small molecule	C29H31N7O3	2020 Approved
Pralsetinib	Gavreto	RET	Capsule	Small molecule	C27H32FN9O2	2020Approved
Capmatinib	Tabrecta	MET	Tablet	Small molecule	C23H17FN6O	2022 Approved
Tepotinib	Tepmetko	MET	Tablet	Small molecule	C29H28N6O2	2021 Approved
Glesatinib		MET	Capsule	Small molecule	C31H27F2N5O3S2	Investigational
Merestinib		MET, ROS1, NTRK	Tablet	Small molecule	C30H22F2N6O3	Investigational
Cabozantinib	Cabometyx	MET	Capsule, Tablet	Small molecule	C28H24FN3O5	Investigational
Trastuzumab deruxtecan	Enhertu	HER2	Injection	Biotech (protein‐based therapies)	Not available	2022 Approved
Larotrectinib	Vitrakvi	NTRK	Capsule, Solution	Small molecule	C21H22F2N6O2	2018 Approved
LY294002		PI3K	Solution	Small molecule	C19H17NO3	Investigational

^1^ The information related to Table 4 was extracted from Drug Bank (go.drugbank.com) and US FDA (fda.gov) databases.

^2^ The status of US FDA approval given in Table 4 was only related to non‐small cell lung cancer (NSCLC).

### 4.2 Combination therapy

Understanding the mechanisms of drug resistance and developing combinational therapies are critical to improving treatment outcomes. This section provides an example of such a cases NSCLC is often treated with a combination of chemotherapy, targeted therapy drugs, or immunotherapy. The special combination of drugs used will depend on the patient's individual case, including the stage and genetic status of their cancer.^370^

Bevacizumab is a targeted drug that has been used in combination with chemotherapy for NSCLC. It is a monoclonal antibody that works by blocking the formation of new blood vessels in tumors, which can help prevent their growth. When used in combination with chemotherapy, bevacizumab has been shown to improve overall survival and progression‐free survival in patients with advanced NSCLC.^371^

Other targeted therapy drugs that have been used in combination with chemotherapy for NSCLC include erlotinib, gefitinib, and afatinib for patients with EGFR mutations, and crizotinib, ceritinib, and alectinib for patients with ALK rearrangements. These drugs target specific genetic mutations found in some NSCLC tumors and can improve response rates and survival outcomes when used in combination with chemotherapy.^372^, ^373^

Ramucirumab in combination with erlotinib has been shown to be an effective first‐line treatment for metastatic NSCLC caused by EGFR (L858R) exon 19 or exon 20 mutations. It is also used in combination with docetaxel for the treatment of metastatic NSCLC in patients who are resistant to platinum‐based chemotherapy.

The combination of dabrafenib and trametinib has also shown a significant effect in metastatic NSCLC patients caused by the BRAFV600E mutation who have not responded to standard treatments.^374^

For most common cancers, chemotherapy is typically included in the main treatment regimen.

Immunotherapy drugs such as pembrolizumab (Keytruda) and atezolizumab (Tecentriq) are often used in combination with chemotherapy. Additionally, osimertinib may be a reasonable option in the treatment of NSCLC, either after the first or second generation of EGFR‐TKI or in combination with pemetrexed or platinum doublet chemotherapy. The LY294002 inhibitor, a PI3K inhibitor, can increase the sensitivity of NSCLC to chemotherapy and radiation therapy. Additionally, miR‐21 has been shown to increase sensitivity to Gefitinib, an EGFR inhibitor, by inhibiting PTEN, PI3K, and AKT pathways in vitro and in vivo.^369^

One other target drug that has shown promise in combination with chemotherapy for NSCLC is pembrolizumab. It is an immunotherapy drug that works by blocking a protein called PD‐1 on the surface of immune cells, which can help the immune system recognize and attack cancer cells. When used in combination with chemotherapy, pembrolizumab has been demonstrated to improve overall survival and progression‐free survival in patients with advanced NSCLC.^375^

TSGs associated with lung cancer include retinoblastoma (RB), tumor protein p53 (TP53), merkel cell carcinoma (MCC), aphidicolin (APH), nonmetastasis 23 (NM23), and APC. The expression of TSGs can effectively inhibit the proliferation and migration of cancer cells and thus regulate tumor progression. Loss of function, knockout, or mutation of TSGs can ultimately lead to tumors. However, TSGs can also be targeted for therapy using CRISPR/Cas9 tools. By using CRISPR technology, these TSGs can be efficiently targeted for repair, leading to the reactivation of TSGs and potentially inhibiting tumor growth.^376^

### 4.3 Clinical trials

Clinical trials are research studies developed to test the safety and efficacy of new treatments for various diseases and conditions. These trials generally consist of several phases, each with a different purpose. Phase I trials are intended to assess the safety of a novel intervention and typically involve a small number of healthy volunteers. Phase II trials are designed to test the effect of a new intervention on a larger group of patients with the disease or condition being studied. Phase III trials are larger and more complex, with the goal of confirming the safety and efficacy of a new intervention in a larger group of patients.

There are currently many clinical trials in progress on compounds for the treatment of NSCLC, investigating various targeted therapies. According to the drugs listed in Table 4, ensartinib is undergoing phase III clinical trials, glesatinib and merestinib are undergoing phase II clinical trials; and LY294002 is undergoing a phase I clinical trial for the treatment of NSCLC.

An example from this section is a phase II clinical trial examining the safety and effectiveness of a combination therapy consisting of Afatinib, which targets EGFR mutations, and nesitumumab, which targets the EGFR pathway, in patients with advanced NSCLC. It should be noted that the US FDA has already approved these two drugs, and their combined use is being tested in a phase II clinical trial.^377^

## 5 CONCLUSIONS

In mammals, methylation plays a significant role in epigenetic modifications, such as tumorigenesis. Aberrant histone modifications have been observed in lung cancer and are believed to contribute to tumor development and progression. Histone modifications are reversible and dynamic changes to histone proteins, that can affect gene expression by altering the accessibility of DNA to transcription factors and other regulatory proteins. In NSCLC, the most common type of lung cancer, decreased levels of the histone modification H3K4me3 have been shown to be associated with poor prognosis and increased tumor invasiveness. Similarly, alterations in histone acetylation have also been observed in lung cancer. Histone acetylation is a vital regulator of gene expression, and aberrant acetylation can lead to dysregulation of genes involved in cell growth and division. In lung cancer, decreased levels of acetylation of histones H3 and H4 have been observed, which is believed to contribute to the development and progression of the disease. Overall, aberrations in histone modifications in lung cancer are an exciting area of research, as they may represent potential targets for therapeutic intervention. By identifying the specific histone modifications that are altered in lung cancer, researchers may be able to develop targeted therapies that can reverse these modifications and improve patient outcomes.

The hypermethylation of CpG islands in the promoter is closely associated with gene inactivation and silencing, which affect the inactivation of TSGs, X‐chromosomes, and cancer development.^82^ Modern diagnostic techniques have been unable to detect most lung cancers at an early stage, and the long‐term prognosis is poor since there are no curative treatments. Therefore, it is crucial to develop and identify biomarkers for early lung cancer diagnosis.^378^ The APC gene is a key TSG; abnormal hypermethylation and mutations in the gene have been affecting most colon cancers and also some other cancers.^136^ It was found by using methylation‐specific PCR that the high percentage of promoter methylation in the *RARβ* and *RASSF1A* genes promotes lung cancer, such that carriers who have aberrant methylation in the promoter of two genes are at a higher risk of lung cancer than carriers of only one gene. This could be a useful marker of prognosis as well as a therapeutic target for lung cancer.^378^ RASSF1A is a major target of tumor‐associated epigenetic dysregulation. It is more commonly silenced by hypermethylation in the promoter and has been closely associated with NSCLC carcinogenesis. Consequently, *RASSF1A* can be known as a suitable putative biomarker in lung cancer.^108^ Moreover, aberrant hypermethylation in the eight genes, including *RASSF1*, *MGMT*, *DAPK1*, *CDH13*, *CDH1*, *RARB*, *KCNH5*, and *BVES*, has been detected in 80% of NSCLC tissues and 14% of noncancerous tissues.^126^

Wnt signaling was initially identified as a crucial pathway for tissue development and homeostasis maintenance. Wnt signaling also has been found to have expanding functions in both solid and liquid tumors, playing a crucial role in modulating the TME and immune response, and highlighting its potential as a target for cancer therapy.^234^ The aberrant activation of Wnt signaling, whether through gene mutations or epigenetic regulation of Wnt signaling component genes, has been closely associated with an increased incidence of cancer malignant progression, potentially leading to an increase in cancer‐related mortality. Additionally, current findings have revealed the importance of Wnt signaling in regulating CSCs, metastasis, and immune control, further highlighting the potential of this pathway as a target for cancer therapy.^234^ Activation of Wnt signaling is related to growing tumor initiation potential, and there is increasing evidence that the Wnt pathway plays vital roles in the development of NSCLC. In Wnt/ß‐catenin signaling pathways, if function in Wnt signaling inhibitors were decreased via promoter hypermethylation or other mechanisms that could be particularly significant in contributing to dedifferentiation, development, tumorigenesis, and prognosis in NSCLC cells. Consequently, restoration of Wnt inhibitor function is associated with increased Wnt signaling, reduced cell proliferation, and reduced apoptosis. In the Wnt signaling pathway, promoter genes with abnormally methylated DNA play significant roles in the Wnt signaling pathway in lung cancer and as a result, cause the aberrant Wnt signaling pathway, which can have a direct impact on tumorigenesis in lung cancer. For example, the APC gene has tumor suppressor activity and an antioncogenic role with regard to Wnt signaling canonical pathways, which are known as one of the main factors that indicate its importance as a tumor suppressor (Table 2). Given its central role in regulating key biological processes, the Wnt signaling pathway has emerged as a critical target for therapeutic interventions aimed at modulating cellular behavior and treating various diseases. Ongoing research into the underlying mechanisms of Wnt signaling promises to deepen our understanding of this pathway's complex functions, providing new insights into its therapeutic potential and opening new avenues for the development of novel treatments.

In the context of cancer, dysregulated expression of miRNAs has been observed in various types of cancer, including lung cancer. This dysregulation can lead to the activation of oncogenes or the suppression of TSGs, which can promote cancer cell growth, invasion, and metastasis. Understanding the dysregulation of miRNA expression in cancer can provide insights into the underlying molecular mechanisms of tumorigenesis, and may lead to the development of novel diagnostic and therapeutic strategies. Several miRNAs have been identified as potential biomarkers for lung cancer diagnosis, such as miR‐21, miR‐210, miR‐155, miR‐126, miR‐486, miR‐205, and miR‐34a.^379^ Additionally, experimental studies have reported that LncRNAs could serve as potential biomarkers for lung cancer due to their crucial roles in controlling several signaling pathways associated with lung tumors. LncRNAs that employ epigenetic mechanisms to modulate a wide range of pathways could be considered significant candidates for diagnostic and prognostic biomarkers related to lung cancer. Thus, targeting miRNAs and LncRNAs has shown promise as a therapeutic strategy for lung cancer. By developing drugs that can specifically target dysregulated miRNAs and LncRNAs, researchers may be able to inhibit tumor growth and improve patient outcomes.^380^ NSCLC accounts for approximately 85% of all lung cancer cases. There are several targeted drugs that have been approved for the treatment of NSCLC, including erlotinib, gefitinib, afatinib, osimertinib, crizotinib, and others. Overall, targeting epigenetic changes is a promising therapeutic approach for the treatment of lung cancer. However, further research is needed to develop more effective and specific drugs that can target epigenetic changes while minimizing off‐target effects.

## AUTHOR CONTRIBUTIONS

S. R. and M. D. were responsible for conducting literature searches related to the topic of the article, while S. R., M. D., Z. S., and A. A. contributed to the writing of the article itself. S. R. was responsible for organizing the structure and framing of the manuscript and also created all of the figures included in the article. A. A. and S. R. edited the final version of the article. Finally, all authors read and approved the final manuscript, indicating their agreement with the content and readiness for publication.

## CONFLICT OF INTEREST STATEMENT

The authors declare no competing interests

## ETHICS STATEMENT

Not applicable.

## Data Availability

Not applicable
